# Supplementary material for: Prevalence of vision impairment among patients with diabetes mellitus in sub-Saharan Africa: A systematic review and meta-analysis
Source: PLoS One. 2025 Jun 24;20(6):e0326176. doi: 10.1371/journal.pone.0326176 (PMC12186915; doi:10.1371/journal.pone.0326176)
Supplement: S2 Text — (DOCX) [file pone.0326176.s002.docx]

**Supplementary information 2: Search strategies**

**Google Scholar:**

Search conducted on 12 November 2023, where possible terms were searched in MeSH as well as text

Record Retrieved=206

allintitle: ("vision loss' OR "visual problem" OR ("vision problem" OR "visual impairment" OR "Vision Disability" OR "Vision Disorder" OR “day Blindness”) AND (diabetes OR "diabetes mellites" OR diabetic OR "Type 1 diabetes" OR "Type 2 diabetes" OR DM) AND [ Country name].

allintitle: (prevalence* OR magnitude OR Epidemiology) AND ("vision loss' OR "visual problem" OR ("vision problem" OR "visual impairment" OR "Vision Disability" OR "Vision Disorder" OR “day Blindness”) AND (diabetes OR "diabetes mellites" OR diabetic OR "Type 1 diabetes" OR "Type 2 diabetes" OR DM) AND [ Country name].

**Search Conducted in Hinari**

Search conducted:13 November 2023

possible terms were searched in MeSH as well as text words

Result Retrieved:23

Summon™: ("vision loss' OR "visual problem" OR ("vision problem" OR "visual impairment" OR "Vision Disability" OR "Vision Disorder" OR “day Blindness”) AND (diabetes OR "diabetes mellites" OR diabetic OR "Type 1 diabetes" OR "Type 2 diabetes" OR DM) AND [ Country name].

Summon™: (prevalence* OR magnitude OR Epidemiology) AND ("vision loss' OR "visual problem" OR ("vision problem" OR "visual impairment" OR "Vision Disability" OR "Vision Disorder" OR “day Blindness”) AND (diabetes OR "diabetes mellites" OR diabetic OR "Type 1 diabetes" OR "Type 2 diabetes" OR DM) AND [ Country name].

**Search Conducted in Medline (PubMed)**

Search conducted on 20 November 2023, where possible terms were searched in MeSH as well as text words

Record Retrieved=21

| **Search** | **Query** | **Records retrieved** |
| --- | --- | --- |
| **#5** | ((((((prevalence[Title/Abstract]) OR (magnitude[Title/Abstract]) OR (Epidemiology[Title/Abstract])) OR (proportion[Title/Abstract])) AND ((((((((((((((((("vision impairment"[Title/Abstract]) OR ("Impairment, Visual"[Title/Abstract])) OR ("Impairment, Visual"[Title/Abstract])) OR ("Visual Impairments"[Title/Abstract])) OR (Micropsia[Title/Abstract])) OR (Micropsias[Title/Abstract])) OR ("Vision Disability"[Title/Abstract])) OR ("Disabilities, Vision"[Title/Abstract])) OR ("Disability, Vision"[Title/Abstract])) OR (Hemeralopia[Title/Abstract])) OR (Day Blindness[Title/Abstract])) OR ("Vision Disorder"[Title/Abstract])) OR ("Visual Disorder"[Title/Abstract])) OR (Macropsia[Title/Abstract])) OR ("Disorder, Visual"[Title/Abstract])) OR ("visual impairment"[Title/Abstract])) OR ("vision problem"[Title/Abstract]))) AND ((((("Diabetes Mellitus"[Title/Abstract]) OR ("Type1 Diabetes Mellitus"[Title/Abstract])) OR ("type 1 diabetes"[Title/Abstract])) OR ("type 2 diabetes"[Title/Abstract])) OR ("Type 2 Diabetes Mellitus"[Title/Abstract]))) AND (((((((((((((((((((((((((((((((((((((((((((((((((((("Sub-Saharan Africa"[Title/Abstract]) OR ("Subsaharan Africa"[Title/Abstract])) OR ("Africa, Sub-Saharan"[Title/Abstract])) OR ("Africa, Central"[Title/Abstract])) OR (Cameroon[Title/Abstract])) OR ("Central African Republic"[Title/Abstract])) OR (Chad[Title/Abstract])) OR (Congo[Title/Abstract])) OR ("Democratic Republic of the Congo"[Title/Abstract])) OR ("Equatorial Guinea"[Title/Abstract])) OR (Gabon[Title/Abstract])) OR ("Sao Tome and Principe"[Title/Abstract])) OR ("Africa, Eastern"[Title/Abstract])) OR (Burundi[Title/Abstract])) OR (Comoros[Title/Abstract])) OR (Djibouti[Title/Abstract])) OR (Eritrea[Title/Abstract])) OR (Ethiopia[Title/Abstract])) OR (Kenya[Title/Abstract])) OR (Madagascar[Title/Abstract])) OR (Rwanda[Title/Abstract])) OR (Seychelles[Title/Abstract])) OR (Somalia[Title/Abstract])) OR (South Sudan[Title/Abstract])) OR (Tanzania[Title/Abstract])) OR (Uganda[Title/Abstract])) OR ("Africa, Southern"[Title/Abstract])) OR (Angola[Title/Abstract])) OR (Botswana[Title/Abstract])) OR (Lesotho[Title/Abstract])) OR (Malawi[Title/Abstract])) OR (Mozambique[Title/Abstract])) OR (Namibia[Title/Abstract])) OR ("South Africa"[Title/Abstract])) OR (Zambia[Title/Abstract])) OR ("Africa, Western"[Title/Abstract])) OR (Benin[Title/Abstract])) OR ("Burkina Faso"[Title/Abstract])) OR ("Cape Verde"[Title/Abstract])) OR ("Cote d'Ivoire"[Title/Abstract])) OR (Gambia[Title/Abstract])) OR (Guinea[Title/Abstract])) OR ("Guinea-Bissau"[Transliterated Title])) OR (Liberia[Title/Abstract])) OR (Mali[Title/Abstract])) OR (Mauritania[Title/Abstract])) OR (Mauritius[Title/Abstract])) OR (Niger[Title/Abstract])) OR (Nigeria[Title/Abstract])) OR (Senegal[Title/Abstract])) OR ("Sierra Leone"[Title/Abstract])) OR (Togo[Title/Abstract])) | 21 |
| **#4** | ((((((((((((((((((((((((((((((((((((((((((((((((((("Sub-Saharan Africa"[Title/Abstract]) OR ("Subsaharan Africa"[Title/Abstract])) OR ("Africa, Sub-Saharan"[Title/Abstract])) OR ("Africa, Central"[Title/Abstract])) OR (Cameroon[Title/Abstract])) OR ("Central African Republic"[Title/Abstract])) OR (Chad[Title/Abstract])) OR (Congo[Title/Abstract])) OR ("Democratic Republic of the Congo"[Title/Abstract])) OR ("Equatorial Guinea"[Title/Abstract])) OR (Gabon[Title/Abstract])) OR ("Sao Tome and Principe"[Title/Abstract])) OR ("Africa, Eastern"[Title/Abstract])) OR (Burundi[Title/Abstract])) OR (Comoros[Title/Abstract])) OR (Djibouti[Title/Abstract])) OR (Eritrea[Title/Abstract])) OR (Ethiopia[Title/Abstract])) OR (Kenya[Title/Abstract])) OR (Madagascar[Title/Abstract])) OR (Rwanda[Title/Abstract])) OR (Seychelles[Title/Abstract])) OR (Somalia[Title/Abstract])) OR (South Sudan[Title/Abstract])) OR (Tanzania[Title/Abstract])) OR (Uganda[Title/Abstract])) OR ("Africa, Southern"[Title/Abstract])) OR (Angola[Title/Abstract])) OR (Botswana[Title/Abstract])) OR (Lesotho[Title/Abstract])) OR (Malawi[Title/Abstract])) OR (Mozambique[Title/Abstract])) OR (Namibia[Title/Abstract])) OR ("South Africa"[Title/Abstract])) OR (Zambia[Title/Abstract])) OR ("Africa, Western"[Title/Abstract])) OR (Benin[Title/Abstract])) OR ("Burkina Faso"[Title/Abstract])) OR ("Cape Verde"[Title/Abstract])) OR ("Cote d'Ivoire"[Title/Abstract])) OR (Gambia[Title/Abstract])) OR (Guinea[Title/Abstract])) OR ("Guinea-Bissau"[Transliterated Title])) OR (Liberia[Title/Abstract])) OR (Mali[Title/Abstract])) OR (Mauritania[Title/Abstract])) OR (Mauritius[Title/Abstract])) OR (Niger[Title/Abstract])) OR (Nigeria[Title/Abstract])) OR (Senegal[Title/Abstract])) OR ("Sierra Leone"[Title/Abstract])) OR (Togo[Title/Abstract]) | 39,0465 |
| **#3** | (((("Diabetes Mellitus"[Title/Abstract]) OR ("Type1 Diabetes Mellitus"[Title/Abstract])) OR ("type 1 diabetes"[Title/Abstract])) OR ("type 2 diabetes"[Title/Abstract])) OR ("Type 2 Diabetes Mellitus"[Title/Abstract]) | 393,501 |
| **#2** | (((((((((((((((("vision impairment"[Title/Abstract]) OR ("Impairment, Visual"[Title/Abstract])) OR ("Impairment, Visual"[Title/Abstract])) OR ("Visual Impairments"[Title/Abstract])) OR (Micropsia[Title/Abstract])) OR (Micropsias[Title/Abstract])) OR ("Vision Disability"[Title/Abstract])) OR ("Disabilities, Vision"[Title/Abstract])) OR ("Disability, Vision"[Title/Abstract])) OR (Hemeralopia[Title/Abstract])) OR (Day Blindness[Title/Abstract])) OR ("Vision Disorder"[Title/Abstract])) OR ("Visual Disorder"[Title/Abstract])) OR (Macropsia[Title/Abstract])) OR ("Disorder, Visual"[Title/Abstract])) OR ("visual impairment"[Title/Abstract])) OR ("vision problem"[Title/Abstract]) | 18,865 |
| **#1** | (((prevalence [Title/Abstract]) OR (magnitude [Title/Abstract])) OR (Epidemiology [Title/Abstract])) OR (proportion [Title/Abstract]) | 1,735,065 |

("vision loss' OR "visual problem" OR ("vision problem" OR "visual impairment" OR "Vision Disability" OR "Vision Disorder" OR “day Blindness”) AND (diabetes OR "diabetes mellites" OR diabetic OR "Type 1 diabetes" OR "Type 2 diabetes" OR DM) AND (“Sub-Saharan Africa” OR “Subsaharan Africa” OR “East Africa” OR “Central Africa” OR Cameroon OR “Central African Republic” OR Chad OR Congo OR “Democratic Republic of the Congo” OR "Equatorial Guinea" OR Gabon OR “Sao Tome and Principe” OR "Africa, Eastern OR Burundi OR Comoros OR Djibouti OR Eritrea OR Ethiopia OR Kenya OR Madagascar OR Rwanda OR Seychelles OR Somalia OR “South Sudan” OR Tanzania OR Uganda OR "Africa, Southern “ OR Angola OR Botswana OR Lesotho OR Malawi OR Mozambique OR Namibia OR "South Africa” OR Zambia OR "Africa, Western” OR Benin OR "Burkina Faso” OR "Cape Verde” OR "Cote d'Ivoire” OR Gambia OR Guinea OR "Guinea-Bissau” OR Liberia OR Mali OR Mauritania OR Mauritius OR Niger OR Nigeria OR Senegal OR "Sierra Leone OR Togo)
