# Supplementary material for: Prevalence of vision impairment among patients with diabetes mellitus in sub-Saharan Africa: A systematic review and meta-analysis
Source: PLoS One. 2025 Jun 24;20(6):e0326176. doi: 10.1371/journal.pone.0326176 (PMC12186915; doi:10.1371/journal.pone.0326176)
Supplement: S3 Table — (DOCX) [file pone.0326176.s003.docx]

**Supplementary file 3: JBI Critical Appraisal Checklist for included Prevalence Studies**

**Reviewer**: WD and ABK, **Date**: December-2023

Yes No Unclear N/A

1. Was the sample frame appropriate

to address the target population? □ □ □

1. Were study participant’s sample

in an appropriate way? □ □ □

1. Was the sample size adequate? □ □ □
2. Were the study subjects and

the setting described in detail? □ □ □

1. Was the data analysis conducted with

sufficient coverage of the identified sample?   □ □ □

1. Were valid methods used for

the identification of the condition?   □ □ □

1. Was the condition measured in

a standard, reliable way for all participants? □ □ □

1. Was there appropriate statistical analysis   □ □ □
2. Was the response rate adequate, and if not, was

the low response rate managed appropriately? □ □ □

Based on these criteria, the quality score for the included studies was given in the following table.

**Table 1**. JBI Critical Appraisal Checklist for the included prevalence studies

| **First Author name, Publication Yr** | **Criteria and corresponding scores** | | | | | | | | | **All over score** |
| --- | --- | --- | --- | --- | --- | --- | --- | --- | --- | --- |
|  | **#1** | **#2** | **#3** | **#4** | **#5** | **#6** | **#7** | **#8** | **#9** |  |
| Seid MA, et al .(2022) | 1 | 1 | 0 | 1 | 1 | 1 | 1 | 1 | 1 | 8 |
| Asemu MT, Ahunie MA. (2021) | 1 | 0 | 1 | 1 | 0 | 1 | 1 | 0 | 1 | 6 |
| Tsegaw A, et al (2021) | 1 | 0 | 1 | 0 | 1 | 1 | 1 | 0 | 1 | 6 |
| Alemu S., Dessie, et al. (2015) | 1 | 1 | 1 | 0 | 1 | 1 | 1 | 1 | 0 | 7 |
| Demilew K .Z, et al. (2022) | 1 | 0 | 1 | 1 | 1 | 0 | 1 | 1 | 1 | 7 |
| Alemayehu H.B, Tegegn et al. (2022) | 1 | 1 | 0 | 0 | 1 | 0 | 1 | 1 | 1 | 6 |
| Bastola et al. (2016) | 0 | 1 | 1 | 1 | 1 | 1 | 1 | 0 | 1 | 7 |
| Glover, Burgess et al. (2012) | 1 | 0 | 0 | 1 | 1 | 0 | 1 | 1 | 1 | 6 |
| Burgess P.I, Allain et al. (2014) | 1 | 1 | 1 | 1 | 1 | 0 | 1 | 0 | 1 | 7 |
| Awadalla. et al (2017) | 0 | 1 | 0 | 1 | 1 | 1 | 1 | 1 | 0 | 6 |
| Sube LK et al.(2020) | 1 | 1 | 1 | 1 | 1 | 1 | 1 | 1 | 0 | 8 |
| Chibuga, Bugimbi.(2012) | 1 | 1 | 1 | 1 | 0 | 1 | 1 | 1 | 1 | 8 |
| Seba EG, Arunga S.(2015) | 1 | 1 | 1 | 1 | 0 | 1 | 1 | 0 | 1 | 8 |
| Magan.T (2019) | 0 | 1 | 0 | 1 | 0 | 1 | 0 | 1 | 1 | 5 |
| Lartey SY, Aikins AK.(2018) | 0 | 1 | 1 | 0 | 1 | 1 | 0 | 1 | 1 | 6 |
| Lewis A.D et al(2018) | 0 | 1 | 1 | 1 | 1 | 0 | 1 | 0 | 1 | 6 |
| Patel.V (2019) | 1 | 0 | 1 | 0 | 1 | 1 | 0 | 1 | 1 | 6 |
| Omari SN .(2017) | 1 | 0 | 1 | 1 | 1 | 1 | 1 | 0 | 1 | 7 |
| Ayukotang E.N et al.(2016) | 1 | 1 | 1 | 0 | 1 | 1 | 1 | 1 | 1 | 8 |
| Jingi, Nansseu et al. (2015) | 0 | 1 | 1 | 1 | 0 | 1 | 1 | 0 | 1 | 6 |
| Onakpoya, Adeoye et al. (2010) | 1 | 1 | 1 | 1 | 1 | 0 | 1 | 0 | 1 | 7 |
| Onakpoya O.H et al (2015) . | 1 | 1 | 1 | 1 | 1 | 0 | 1 | 1 | 0 | 7 |
| Sada B.K et al( 2021) | 1 | 1 | 1 | 0 | 1 | 1 | 0 | 1 | 0 | 6 |
| Ajayi. I.A(2016) | 1 | 1 | 1 | 1 | 1 | 1 | 1 | 0 | 0 | 7 |
| Cleland, Charles R., et al. (2016) | 1 | 1 | 1 | 0 | 1 | 0 | 1 | 0 | 1 | 6 |
| Mabaso and Oduntan. (2014) | 1 | 1 | 1 | 0 | 1 | 0 | 0 | 1 | 1 | 6 |

**NB**: **“1”** indicates the article does fulfill the specified criteria, and **“0”** indicates the article does not fulfill the stated criteria
