# Supplementary material for: Prevalence of vision impairment among patients with diabetes mellitus in sub-Saharan Africa: A systematic review and meta-analysis
Source: PLoS One. 2025 Jun 24;20(6):e0326176. doi: 10.1371/journal.pone.0326176 (PMC12186915; doi:10.1371/journal.pone.0326176)
Supplement: S4 Table — (DOCX) [file pone.0326176.s004.docx]

**Table 1: Description of Visual Acuity Measurement for the included studies**

| **First author Name, Year** | **Country** | **Visual impairment definition** | **Types of VA Measurements** | **Method/ tool used for VA testing** | **Grouping/classification of VI** |
| --- | --- | --- | --- | --- | --- |
| Seid MA, *et al*. (2022) | Ethiopia | VI: VA worse than 6/18 (<6/18) | PVA | Tumbling-E Snellen’s charts at 6m distance | WHO classification of VA |
| Asemu MT, Ahunie MA (2021) | Ethiopia | VI: VA < 6/18 | PVA | Projection charts at 6m distance | WHO classification of VA |
| Tsegaw A , *et al.* (2021) | Ethiopia | VI: VA- worse than 6/18 in the better eye | BCVA | Tumbling-E Snellen’s charts | WHO classification of VA |
| Alemu S., Dessie*, et al*. (2015) | Ethiopia | VI: VA worse than 6/18 in the better eye | BCVA | Tumbling-E Snellen’s charts | WHO classification of VA |
| Demilew K .Z, *et al*. (2022) | Ethiopia | VI: VA worse than 6/18 (<6/18) | PVA | Tumbling-E Snellen’s charts | WHO classification of VA |
| Alemayehu H.B, Tegegn, *et al*. (2022) | Ethiopia | VI: VA < 6/12 in the better eye. | PVA | Snellen chart at 6 meters distance | ICD-11 definition |
| Bastola, *et al.* (2016) | Eritrea | VI: VA < 6/18 in the better eye | BCVA | Not mentioned | WHO classification of VA |
| Glover, Burgess, *et al*. (2012) | Malawi | VI: VA worse than 6/18 in the better eye | BCVA | Snellen chart | WHO classification of VA |
| Burgess P.I, Allain, *et al*. (2014) | Malawi | VI: <60 ETDRS letters (approx. worse than 6/18 in better eye) | BCVA | Early Treatment Diabetic Retinopathy Study (ETDRS) chart/ or Tumbling E logMAR chart at 4 meters with pinhole test | WHO classification of VA |
| Awadalla (2017) | Sudan | Not specified | Not specified | Not specified | Not specified |
| Sube LK,*et al*.(2020) | South Sudan | VI: VA < 6/18 in the better eye | BCVA | Snellen chart with /without pinhole test | WHO classification of VA |
| Chibuga, Bugimbi. (2012) | Tanzania | VI: VA < 6/18 of the best eye | Not specified | Snellen’s E chart at 6 m | WHO classification of VA |
| Seba EG, Arunga S.(2015) | Uganda | VI: VA worse than 6/18 in the better eye | BCVA | Snellen’s E chart at 6 m | WHO classification of VA |
| Magan.T (2019) | Uganda | VI: VA< 6/18 in the better eye | BCVA | Snellen chart at 6m | WHO classification of VA |
| Lartey SY, Aikins AK.(2018) | Ghana | VI: VA worse than 6/18 in the better eye | BCVA | Snellen acuity chart | WHO classification of VA |
| Lewis A.D*, et al*.(2018) | Zambia | VI: VA worse than 6/18 in the better eye | BCVA | Snellen chart at 6 m | WHO definitions |
| Patel.V. (2019) | Zambia | VI: VA worse than 6/12 | BCVA | Snellen chart | ICD-11 classification of VA |
| Omari S.N(2017) | Botswana | VA: VA worse than 6/18 in the better eye | BCVA | Snellen chart with pinhole testing | WHO classification of VA |
| Ayukotang E.N, *et a*l.(2016) | Cameroon | VI: VA worse than 6/18 in the better eye | Not specified | Snellen VA chart | WHO classification of VA |
| Jingi, Nansseu, *et al*. (2015) | Cameron | VI: VA < 3/10 | BCVA | Projection chart at 6m didtance | International Council of Ophthalmology (ICO) |
| Onakpoya, Adeoye, *et al*. (2010) | Nigeria | VI: VA < 6/18 in the better eye | BCVA | Snellen’s or Tumbling E chart at a 6m distance using a pinhole/ spectacle | WHO classification of VA |
| Onakpoya. O H, *et al*. (2015) | Nigeria | VI: VA < 6/18 in the better eye | BCVA | Snellen’s chart or Tumbling E chart at a 6m distance using a pinhole test and/or patient's glasses | WHO classification of VA |
| Sada B.K et al(2021) | Nigeria | Not specified | Not specified | Not specified | Not specified |
| Ajayi. I.A(2016) | Nigeria | VI: VA < 6/18 in the better eye | PVA | Not specified | WHO classification of VA |
| Cleland, Charles R., *et al*. (2016) | Tanzania | VI: VA < 6/18 in the better eye | Not specified | Not specified | WHO classification of VA |
| Mabaso and Oduntan. (2014) | South Africa | VI: VA < 6/9.5 in the better eye | BCVA | LogMAR VA Chart with /or without pinhole test |  |

**Abbreviations**: VI= Visual impairment, VA= Visual Acuity, PVA= Presenting Visual Acuity, BCVA= Best Corrected Visual Acuity.
