# Supplementary material for: Prevalence of vision impairment among patients with diabetes mellitus in sub-Saharan Africa: A systematic review and meta-analysis
Source: PLoS One. 2025 Jun 24;20(6):e0326176. doi: 10.1371/journal.pone.0326176 (PMC12186915; doi:10.1371/journal.pone.0326176)
Supplement: S5 Fig — (DOCX) [file pone.0326176.s005.docx]

Supplementary file 5. Sensitivity analysis for the studies included in prevalence of Visual impairment (VI) among diabetes

## Fig.1. Sensitivity analysis of prevalence for each study being removed at a time: Prevalence and 95% CI of VI among diabetes in SSA.
