# Supplementary material for: Prevalence of vision impairment among patients with diabetes mellitus in sub-Saharan Africa: A systematic review and meta-analysis
Source: PLoS One. 2025 Jun 24;20(6):e0326176. doi: 10.1371/journal.pone.0326176 (PMC12186915; doi:10.1371/journal.pone.0326176)
Supplement: S6 Fig — (DOCX) [file pone.0326176.s006.docx]

Supplementary file 5: Forest plot showing causes of visual impairment among patients with diabetes mellites, in Sab-Saharan Africa.


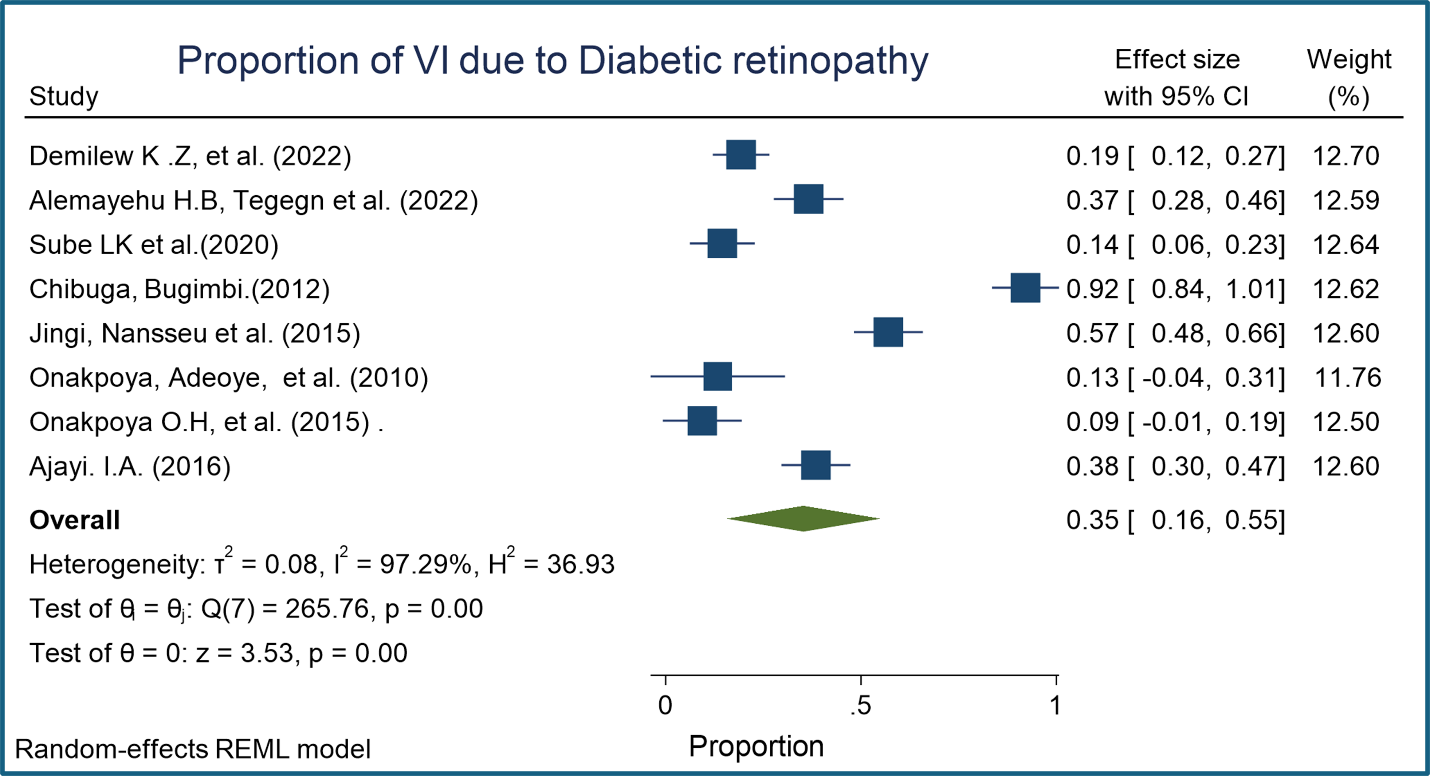


Fig 1. Forest plot showing the pooled proportion of visual impairment (VI) due to diabetic retinopathy among patients with diabetes mellites, in Sab-Saharan Africa,2023.

Fig 2. Forest plot showing the pooled proportion of visual impairment (VI) due to diabetic maculopathy among patients with diabetes mellites, in sub-Saharan Africa,2023.

Fig 3. Forest plot showing the pooled proportion of visual impairment (VI) due to cataract among patients with diabetes mellites, in sub-Saharan Africa,2023.

Fig 2. Forest plot showing the pooled proportion of visual impairment (VI) due to glaucoma among patients with diabetes mellites, in sub-Saharan Africa,2023

Fig 2. Forest plot showing the pooled proportion of visual impairment (VI) due to macular degeneration among patients with diabetes mellites, in sub-Saharan Africa,2023

Fig 2. Forest plot showing the pooled proportion of visual impairment (VI) due to refractory error among patients with diabetes mellites, in sub-Saharan Africa,2023

Fig 2. Forest plot showing the pooled proportion of visual impairment (VI) due to *other causes among patients with diabetes mellites, in sub-Saharan Africa,2023

*Other causes = hypertensive retinopathy, Dry eye syndrome, Pseudophakia, ocular infection
