# Supplementary material for: Prevalence of vision impairment among patients with diabetes mellitus in sub-Saharan Africa: A systematic review and meta-analysis
Source: PLoS One. 2025 Jun 24;20(6):e0326176. doi: 10.1371/journal.pone.0326176 (PMC12186915; doi:10.1371/journal.pone.0326176)
Supplement: S9 Table — (DOCX) [file pone.0326176.s009.docx]

**Supplementary File 7**

Table. List of all studies identified in the literature search, including those that were excluded from the analyses.

| **S.N** | **List of studies** | **Remark** | | |
| --- | --- | --- | --- | --- |
|  |  | Included | Excluded | Reason(s) for exclusion |
|  | Hussain, Asim, et al. "A Comprehensive Review on Diabetic Retinopathy and Mental Disorders." *IOSR Journal of Biotechnology and Biochemistry* 6.3 (2020): 38-51 |  | √ | -Review article |
|  | Fentie, Dilnessa, Yonatan Solomon, and Tameru Menberu. "The burden of visual impairment among Ethiopian adult population: Systematic review and meta-analysis." *PloS one* 18.7 (2023): e0288707. |  |  | -Review Article |
|  | Abejew AA, Belay AZ, Kerie MW. Diabetic complications among adult diabetic patients of a tertiary hospital in northeast Ethiopia. Advances in Public Health. 2015;2015(1):290920. |  | √ | -Outcome of interest not reported |
|  | Abikoye TM, Aribaba OT, Musa KO, Idowu OO. Prevalence and causes of visual impairment among hearing impaired students in Lagos, Nigeria. International journal of pediatric otorhinolaryngology. 2020;139:110487. |  | √ | -Non-Diabetic Study participants |
|  | Abonyi MC, Ugwu TE, Eze CC. Prevalence and risk factors for Diabetic Retinopathy in a tertiary institution in South Eastern Nigeria. Annals of Clinical and Biomedical Research. 2023;4(2). |  | √ | -Outcome of interest not reported |
|  | Abraham E, Umoh V. Prevalence of diabetic retinopathy in diabetes mellitus patients attending a tertiary eye clinic in Uyo south-south Nigeria. Ibom Medical Journal. 2013;6(2):1-7. |  | √ | -Outcome of interest not reported |
|  | Abraham EG, Umoh V. Ocular Health Status of Diabetes Mellitus Patients in Uyo, South-South Nigeria. IOSR Journal of Dental and Medical Sciences. 2013;9(2):24-8. |  | √ | -Outcome of interest not reported |
|  | Abubakar T, Gudlavalleti MV, Sivasubramaniam S, Gilbert CE, Abdull MM, Imam AU. Coverage of hospital-based cataract surgery and barriers to the uptake of surgery among cataract blind persons in nigeria: the Nigeria National Blindness and Visual Impairment Survey. Ophthalmic epidemiology. 2012;19(2):58-66. |  | √ | -Unrelated title |
|  | Adebusoye LA, Owoaje ET, Ladipo MM, Adeniji AO. Visual morbidities among elderly patients presenting at a primary care clinic in Nigeria. West African journal of medicine. 2011;30(2):118-20. |  | √ | -Unrelated title |
|  | Bekele, Bayu Begashaw. "The prevalence of macro and microvascular complications of DM among patients in Ethiopia 1990–2017: Systematic review." *Diabetes & Metabolic Syndrome: Clinical Research & Reviews* 13.1 (2019): 672-677. |  | √ | -Review article |
|  | Agweye CT, Udoh M-ME, Etim BA, Ibanga AA, Nkanga ED, Nkanga DG, et al. Risk factors for diabetic retinopathy in patients with type 2 diabetes mellitus. A hospital-based study. Nigerian Journal of Vitreoretinal Diseases. 2023;6(2):25-30. |  | √ | --Outcome of interest not reported |
|  | Ajayi IA, Raimi TH, Omotoye OJ, Ajite K. Ocular findings in a diabetic retinopathy screening clinic in Southwest Nigeria. Sky J Med Med Sci. 2016;4:23-7. |  | √ | -Outcome of interest not reported |
|  | Akudinobi CU, SN NN. Prevalence of visual impairment among the destitute in Onitsha, Southern Nigeria. Nigerian journal of clinical practice. 2022;25(8):1211-5. |  | √ | -Non-Diabetic Study participants |
|  | Alemu Mersha G, Alimaw YA, Woredekal AT. Prevalence of diabetic retinopathy among diabetic patients in Northwest Ethiopia-A cross sectional hospital based study. PloS one. 2022;17(1):e0262664. |  | √ | -Unclear methodology  -Outcome of interest not reported |
|  | Aliyu R, Gezawa ID, Uloko AE, Ramalan MA. Prevalence and risk factors of diabetes foot ulcers in Kano, northwestern Nigeria. Clinical Diabetes and Endocrinology. 2023;9(1):6. |  | √ | -Unrelated title |
|  | Amiebenomo OM, Isong EM, Edosa ME, Woodhouse JM. Habitual visual acuity and visual acuity threshold demands in Nigerian school classrooms. Scientific reports. 2022;12(1):17816. |  | √ | -Unrelated title |
|  | Asimadu IN, Kizor-Akaraiwe N, Ezegwui I, Okeke S. Ocular morbidity among persons with diabetes in Enugu, Nigeria. SN Comprehensive Clinical Medicine. 2020;2:782-7. |  | √ | -Unclear methodology  -Outcome of interest not reported |
|  | Ruta LM, Magliano DJ, Lemesurier R, Taylor HR, Zimmet PZ, Shaw JE. Prevalence of diabetic retinopathy in Type 2 diabetes in developing and developed countries. Diabetic medicine. 2013 Apr;30(4):387-98. |  | √ | -Review article |
|  | Association AD. 10. Microvascular complications and foot care: standards of medical care in diabetes—2018. Diabetes care. 2018;41(Supplement_1):S105-S18. |  | √ | -Outcome of interest not reported |
|  | Awadalla H, Noor SK, Elmadhoun WM, Almobarak AO, Elmak NE, Abdelaziz SI, et al. Diabetes complications in Sudanese individuals with type 2 diabetes: Overlooked problems in sub-Saharan Africa? Diabetes & Metabolic Syndrome: Clinical Research & Reviews. 2017;11:S1047-S51. |  | √ | -Outcome of interest not reported |
|  | Ibrahim, Amin Muhammad. "Epidemiological Review of Chronic Diabetes Complications (Cardiovascular Disease, Nephropathy and Retinopathy)." *EC Endocrinol Metab Res* 3 (2018): 103-113.. |  | √ | -Review article |
|  | Aziz K. Association of Diabetic Retinopathy and Maculopathy with Elevated HbA1c. Blood Pressure, Serum Creatinine, Microalbuminuria, Spot Urine Protein, Nephropathy and Diabetic Kidney Disease An Experience from Data Analysis of. 2018;10:1-11. |  | √ | -Outcome of interest not reported |
|  | Azizi-Soleiman F, Heidari-Beni M, Ambler G, Omar R, Amini M, Hosseini S-M. Iranian risk model as a predictive tool for retinopathy in patients with type 2 diabetes. Canadian journal of diabetes. 2015;39(5):358-63. |  | √ | -Study conducted outside SSA |
|  | Babalola O. The peculiar challenges of blindness prevention in Nigeria: a review article. 2011. |  | √ | Review article |
|  | Babalola OE. The peculiar challenges of blindness prevention in Nigeria: a review article. African journal of medicine and medical sciences. 2011;40(4):309-19. |  | √ | - Review article |
|  | Bamashmus MA, Gunaid AA, Khandekar RB. Diabetic retinopathy, visual impairment and ocular status among patients with diabetes mellitus in Yemen: a hospital-based study. Indian journal of ophthalmology. 2009;57(4):293-8. |  | √ | -Study conducted outside SSA |
|  | Bastawrous A, Mathenge W, Wing K, Bastawrous M, Rono H, Weiss HA, et al. The incidence of diabetes mellitus and diabetic retinopathy in a population-based cohort study of people age 50 years and over in Nakuru, Kenya. BMC endocrine disorders. 2017;17:1-14. |  | √ | -Outcome of interest not reported |
|  | Bastawrous A, Mathenge W, Wing K, Rono H, Gichangi M, Weiss HA, et al. Six-Year Incidence of Blindness and Visual Impairment in Kenya: The Nakuru Eye Disease Cohort Study. Investigative ophthalmology & visual science. 2016;57(14):5974-83. |  | √ | -Non-Diabetic Study participants |
|  | Bhaskaranand M, Ramachandra C, Bhat S, Cuadros J, Nittala MG, Sadda SR, et al. The value of automated diabetic retinopathy screening with the EyeArt system: a study of more than 100,000 consecutive encounters from people with diabetes. Diabetes technology & therapeutics. 2019;21(11):635-43. |  | √ | -Outcome of interest not reported |
|  | Bhavana N. Study of Progression of Diabetic Retinopathy Following Small Incision Cataract Surgery: Rajiv Gandhi University of Health Sciences (India); 2019. |  | √ | -Study conducted outside SSA |
|  | Blake AM, Munby HN, Katlego PM, Sebuyuyu PM, Nkomozana O, Bangure R, et al. Characteristics of patients with diabetic retinopathy in Gaborone, Botswana. Tanzania Journal of Health Research. 2015;17(1). |  | √ | -Outcome of interest not reported |
|  | Bowen M, Edgar DF, Hancock B, Haque S, Shah R, Buchanan S, et al. The Prevalence of Visual Impairment in People with Dementia (the PrOVIDe study): a cross sectional study of 60-89 year old people with dementia and qualitative exploration of individual, carer and professional perspectives. Health and Social Care Delivery Research. 2016;4(21):1-200. |  | √ | -Non-Diabetic Study participants |
|  | Branislava I, Editat S, Zoran S, Obradovic M, Bozidarkas ZL, Isenovic ER. and Dysfunctional Endothelial Phenotype in Obesity. Bioactive Food as Dietary Interventions for Diabetes. 2019:231. |  | √ | -Unrelated title |
|  | Burgess PI. Determinants of severity and progression of diabetic retinopathy in Southern Malawi: The University of Liverpool (United Kingdom); 2015. |  | √ | --Unrelated title |
|  | Burgess PI, Harding SP, García-Fiñana M, Beare NA, Glover S, Cohen DB, et al. Incidence and progression of diabetic retinopathy in Sub-Saharan Africa: A five year cohort study. PloS one. 2017;12(8):e0181359. |  | √ | -Unclear methodology |
|  | Burgess PI, Harding SP, García-Fiñana M, Beare NA, Msukwa G, Allain TJ. First prospective cohort study of diabetic retinopathy from sub-Saharan Africa: high incidence and progression of retinopathy and relationship to human immunodeficiency virus infection. Ophthalmology. 2016;123(9):1919-25. |  | √ | ---Unrelated title |
|  | Burgess PI, Msukwa G, Beare NA. Diabetic retinopathy in sub-Saharan Africa: meeting the challenges of an emerging epidemic. BMC medicine. 2013;11:1-7. |  | √ | --Unrelated title |
|  | Azeez, Taoreed Adegoke, et al. "Prevalence and risk factors for diabetic retinopathy in Nigeria: A systematic review and meta-analysis." *The Pan-American Journal of Ophthalmology* 3.1 (2021): 17. |  | √ | -Review article |
|  | Cacchione PZ. Sensory changes. Evidence‐based geriatric nursing pro‐tocols for best practice. 2011:48-74. |  | √ | -Unrelated title |
|  | Cairncross JP, Steinberg WJ, Labuschagne MJ. Prevalence of eye pathology in a group of diabetic patients at National District Hospital Outpatient Department in Bloemfontein, South Africa. African Journal of Primary Health Care and Family Medicine. 2017;9(1):1-7. |  | √ | -Unclear methodology |
|  | Care D. Medical care in diabetes 2018. Diabet Care. 2018;41(1):S105-S18. |  | √ | -unrelated title |
|  | White, Neil H. "Long-term outcomes in youth with diabetes mellitus." *Pediatric Clinics of North America* 62.4 (2015): 889. |  | √ | -Review article |
|  | Chowdhury IA. Prevalence of epidemiological influence, risk factors of type-2 Diabetes Mellitus and analysis of hypertension as a complication among the relatively newly diagnosed patients from BIRDEM hospital: BRAC University; 2017. |  | √ | --Study conducted outside SSA |
|  | Chua J, Lim CXY, Wong TY, Sabanayagam C. Diabetic retinopathy in the Asia-Pacific. The Asia-Pacific Journal of Ophthalmology. 2018;7(1):3-16. |  | √ | -Study conducted outside SSA |
|  | Marques, Ana Patricia et al. “The economics of vision impairment and its leading causes: A systematic review.” *EClinicalMedicine* vol. 46 101354. 22 Mar. 2022, doi:10.1016/j.eclinm.2022.101354 |  | √ | -Review article |
|  | Cui J, Ren J-P, Chen D-N, Xin Z, Yuan M-X, Xu J, et al. Prevalence and associated factors of diabetic retinopathy in Beijing, China: a cross-sectional study. BMJ open. 2017;7(8):e015473. |  | √ | -Study conducted outside SSA |
|  | Cui Y, Zhang L, Zhang M, Yang X, Zhang L, Kuang J, et al. Prevalence and causes of low vision and blindness in a Chinese population with type 2 diabetes: the Dongguan Eye Study. Scientific reports. 2017;7(1):11195. |  | √ | -Study conducted outside SSA |
|  | Cui Y, Zhang M, Zhang L, Zhang L, Kuang J, Zhang G, et al. Prevalence and risk factors for diabetic retinopathy in a cross-sectional population-based study from rural southern China: Dongguan Eye Study. BMJ open. 2019;9(9):e023586. |  | √ | -Study conducted outside SSA |
|  | Cushley L, Hageman G. The role of the peripheral retina in diabetic retinopathy: from basic science to town planning: Queen's University Belfast; 2023. |  | √ | -Unrelated title |
|  | Debesai M, Russom M. Praziquantel and risk of visual disorders: Case series assessment. PLoS neglected tropical diseases. 2020;14(4):e0008198. |  | √ | -Unrelated title |
|  | Diress M, Belsti Y, Getnet M, Fekadu SA, Dagnew B, Akalu Y, et al. Visual impairment and associated factors among pregnant women attending antenatal care units at health institutions in Gondar City Administration, Northwest Ethiopia. BMC pregnancy and childbirth. 2021;21(1):824. |  | √ | -Non-Diabetic Study participants |
|  | Duke R, Otong E, Iso M, Okorie U, Ekwe A, Courtright P, et al. Using key informants to estimate prevalence of severe visual impairment and blindness in children in Cross River State, Nigeria. Journal of AAPOS : the official publication of the American Association for Pediatric Ophthalmology and Strabismus. 2013;17(4):381-4. |  | √ | -Unrelated title |
|  | E JY, Wang Z, Ssekasanvu J, Munoz B, West S, Ludigo J, et al. Visual Impairment and Eye Diseases in HIV-infected People in the Antiretroviral Therapy (ART) Era in Rakai, Uganda. Ophthalmic epidemiology. 2021;28(1):63-9. |  | √ | -Non-diabetic study participant |
|  | Egunsola O, Dowsett LE, Diaz R, Brent MH, Rac V, Clement FM. Diabetic retinopathy screening: a systematic review of qualitative literature. Canadian journal of diabetes. 2021;45(8):725-33. e12. |  | √ | - Review article |
|  | Ehrlich JR, Stagg BC, Andrews C, Kumagai A, Musch DC. Vision Impairment and Receipt of Eye Care Among Older Adults in Low- and Middle-Income Countries. JAMA ophthalmology. 2019;137(2):146-58. |  | √ | -Non-diabetic study participant |
|  | Ejiakor I, Achigbu E, Onyia O, Edema O, Florence UN. Impact of Visual Impairment and Blindness on Quality of Life of Patients in Owerri, Imo State, Nigeria. Middle East African journal of ophthalmology. 2019;26(3):127-32. |  | √ | -Unrelated title |
|  | Ejiakor IL, Achigbu EO, Onyia OE, Edema O, Nkwogu FU, Okeke AJ, et al. Comparative Analysis of the Impact of Visual Impairment on Quality of Life of Patients Attending a Tertiary Hospital in South East, Nigeria. Nigerian journal of clinical practice. 2022;25(1):5-11. |  | √ | -Unrelated title |
|  | EK MPRKB, Kohner KEM. Saving Sight: A History of Diabetic Eye Disease. Unveiling Diabetes-Historical Milestones in Diabetology. 2020:221. |  | √ | -Unrelated title |
|  | Elwali ES, Almobarak AO, Hassan MA, Mahmooud AA, Awadalla H, Ahmed MH. Frequency of diabetic retinopathy and associated risk factors in Khartoum, Sudan: population based study. International journal of ophthalmology. 2017;10(6):948. |  | √ | -Unrelated title |
|  | Jonas, Jost B et al. “Visual impairment and blindness due to macular diseases globally: a systematic review and meta-analysis.” *American journal of ophthalmology* vol. 158,4 (2014): 808-15. doi:10.1016/j.ajo.2014.06.012 |  | √ | -Review article |
|  | Ephraim RKD, Anoff KA, Brenyah RC, Osakunor DNM, Sakyi SA, Osei-Yeboah J, et al. Determinants of crystalluria among type 2 diabetes patients; A case-control study of the Agona West Municipality, Ghana. Nigerian Medical Journal. 2017;58(3):114-8. |  | √ | -Unrelated title |
|  | Ewuga RO, Adenuga OO, Wade PD, Edah JO. Prevalence and risk factors for diabetic retinopathy in north-central Nigeria. Ghana Medical Journal. 2018;52(4):215-21. |  | √ | -Unclear methodology |
|  | Fadamiro CO. Causes of blindness and career choice among pupils in a blind school; South Western Nigeria. Annals of African medicine. 2014;13(1):16-20. |  | √ | -Unrelated title |
|  | Feldman-Billard S, Dupas B. Eye disorders other than diabetic retinopathy in patients with diabetes. Diabetes & Metabolism. 2021;47(6):101279. |  | √ | -Outcome of interest not reported |
|  | Fenwick E, Rees G, Pesudovs K, Dirani M, Kawasaki R, Wong TY, et al. Social and emotional impact of diabetic retinopathy: a review. Clinical & experimental ophthalmology. 2012;40(1):27-38. |  | √ | - Review article |
|  | Fenwick EK, Pesudovs K, Khadka J, Rees G, Wong TY, Lamoureux EL. Evaluation of item candidates for a diabetic retinopathy quality of life item bank. Quality of Life Research. 2013;22:1851-8. |  | √ | -Unrelated title |
|  | Fife B. Stop Vision Loss Now!: Prevent and Heal Cataracts, Glaucoma, Macular Degeneration, and Other Common Eye Disorders: Piccadilly Books, Ltd.; 2017. |  | √ | -Unrelated title |
|  | Fite RO, Lake EA, Hanfore LK. Diabetic retinopathy in Ethiopia: a systematic review and meta-analysis. Diabetes & Metabolic Syndrome: Clinical Research & Reviews. 2019;13(3):1885-91. |  | √ | - Review article |
|  | Gale R, Scanlon PH, Evans M, Ghanchi F, Yang Y. and Silvestri, G and Freeman, M and Maisey, A and Napier, J (2017) Action on diabetic macular oedema: achieving optimal patient management in treating visual impairment due to diabetic eye disease. Eye, 31 (S1). S1-S20. ISSN 0950-222X. Eye. 2017;31:S1-S20. |  | √ | -Unrelated title |
|  | Gascoyne B, Jolley E, Penzin S, Ogundimu K, Owoeye F, Schmidt E. Vision impairment and self-reported anxiety and depression in older adults in Nigeria: evidence from a cross-sectional survey in Kogi State. International health. 2022;14(Suppl 1):i9-i16. |  | √ | -Unrelated title |
|  | Gbessemehlan A, Edjolo A, Helmer C, Delcourt C, Mbelesso P, Ndamba-Bandzouzi B, et al. Vision Impairment and Adverse Health Conditions in Congolese Older People: A Population-Based Study. Gerontology. 2022;68(4):387-96. |  | √ | -Non-diabetic study participant |
|  | Kahloun, Rim et al. “Prevalence and causes of vision loss in North Africa and Middle East in 2015: magnitude, temporal trends and projections.” *The British journal of ophthalmology* vol. 103,7 (2019): 863-870. doi:10.1136/bjophthalmol-2018-312068 |  | √ | -Review article |
|  | Gebremariam MG, Bacha RH, Demissie DK, Wolde KS, Dame KT, Akessa GM. Modeling Time to Blindness of Glaucoma Patients: A Case Study at Jimma University Medical Center. Journal of research in health sciences. 2022;22(2):e00548. |  | √ | -Unrelated title |
|  | Oluleye, T S. “Diabetic retinopathy: current developments in pathogenesis and management.” *African journal of medicine and medical sciences* vol. 39,3 (2010): 199-206. |  | √ | -Review article |
|  | Ghaem H, Daneshi N, Riahi S, Dianatinasab M. The prevalence and risk factors for diabetic retinopathy in Shiraz, Southern Iran. Diabetes & metabolism journal. 2018;42(6):538-43. |  | √ | -Study conducted outside SSA |
|  | Giloyan A, Muradyan D, Khachadourian V. Visual impairment and associated risk factors in patients with diabetes mellitus in Tavush and Armavir provinces of Armenia. International ophthalmology. 2022;42(1):47-56. |  | √ | -Study conducted outside SSA |
|  | Haddad NMN, Sun JK, Abujaber S, Schlossman DK, Silva PS, editors. Cataract surgery and its complications in diabetic patients. Seminars in ophthalmology; 2014: Taylor & Francis. |  | √ | -Unrelated title |
|  | Hajar S, Al Hazmi A, Wasli M, Mousa A, Rabiu M. Prevalence and causes of blindness and diabetic retinopathy in Southern Saudi Arabia. Saudi medical journal. 2015;36(4):449. |  | √ | -Unclear methodology |
|  | Hall C, Hall A, Kok G, Mallya J. A health care worker needs assessment to develop rural diabetic training workshops for diabetes and diabetic retinopathy screening in Kilimanjaro. Using intervention mapping to establish a rural screening program for diabetic retinopathy in Kilimanjaro, Northern Tanzania. 2023:51. |  | √ | -Unrelated title |
|  | Hayreh SS. Management of ischemic optic neuropathies. Indian journal of ophthalmology. 2011;59(2):123-36. |  | √ | -Unrelated title |
|  | He B-B, Wei L, Gu Y-J, Han J-F, Li M, Liu Y-X, et al. Factors associated with diabetic retinopathy in chinese patients with type 2 diabetes mellitus. International journal of endocrinology. 2012;2012(1):157940. |  | √ | -Study conducted outside SSA |
|  | Hernández-Moreno L, Senra H, Moreno N, Macedo AF. Is perceived social support more important than visual acuity for clinical depression and anxiety in patients with age-related macular degeneration and diabetic retinopathy? Clinical Rehabilitation. 2021;35(9):1341-7. |  | √ | --Unrelated title |
|  | Heward J, Stone L, Paddick SM, Mkenda S, Gray WK, Dotchin CL, et al. A longitudinal study of cognitive decline in rural Tanzania: rates and potentially modifiable risk factors. International psychogeriatrics. 2018;30(9):1333-43. |  | √ | -Unrelated title |
|  | Heydari B, Yaghoubi G, Yaghoubi MA, Miri MR. Prevalence and risk factors for diabetic retinopathy: an Iranian eye study. European Journal of Ophthalmology. 2012;22(3):393-7. |  | √ | -Study conducted outside SSA |
|  | Hien H, Berthé A, Drabo MK, Meda N, Konaté B, Tou F, et al. Prevalence and patterns of multimorbidity among the elderly in Burkina Faso: cross-sectional study. Tropical medicine & international health : TM & IH. 2014;19(11):1328-33. |  | √ | -Unrelated title |
|  | Hinju P. Diabetic Retinopathy and Associated Factors in Patients with Diabetes Mellitus at Muhimbili National Hospital: Muhimbili University of Health and Allied Sciences; 2021. |  | √ | -Unclear methodology |
|  | Hirpesa GM. Ophthalmology Care in Ethiopia: a Health Economic Evaluation: The University of Bergen; 2022. |  | √ | -Unrelated title |
|  | Hofman KJ, Cook C, Levitt N. Preventing diabetic blindness: a priority for South Africa. South African medical journal = Suid-Afrikaanse tydskrif vir geneeskunde. 2014;104(10):661-2. |  | √ | -Unrelated title |
|  | Hope M. Assessing the Knowledge and Practices regarding eye care and complications of Diabetes among Diabetic Patients 18 years and older, attending a tertiary Diabetic Clinic in Kampala, Uganda. 2019. |  | √ | -Outcome of interest not reported |
|  | Horri N, Farmani M, Ghassami M, Haghighi S, Amini M. Visual acuity in an Iranian cohort of patients with type 2 diabetes: the role of nephropathy and ischemic heart disease. Journal of Research in Medical Sciences: the Official Journal of Isfahan University of Medical Sciences. 2011;16(Suppl1):S419. |  | √ | -Study conducted outside SSA |
|  | Hou X, Wang L, Zhu D, Guo L, Weng J, Zhang M, et al. Prevalence of diabetic retinopathy and vision-threatening diabetic retinopathy in adults with diabetes in China. Nature Communications. 2023;14(1):4296. |  | √ | -Study conducted outside SSA |
|  | Hydara A, Mactaggart I, Bell SJ, Okoh JA, Olaniyan SI, Aleser M, et al. Prevalence of blindness and distance vision impairment in the Gambia across three decades of eye health programming. The British journal of ophthalmology. 2023;107(6):876-82. |  | √ | -Non-diabetic study participant |
|  | Javaloy J, Moya T, Muñoz G, Albarrán-Diego C, Valls-Martínez J, Montalbán R, et al. Efficacy, safety and visual outcomes of cataract surgeries performed during blindness prevention programs in different locations in Kenya. Graefe's archive for clinical and experimental ophthalmology = Albrecht von Graefes Archiv fur klinische und experimentelle Ophthalmologie. 2021;259(5):1215-24. |  | √ | -Unrelated title |
|  | Jivraj I, Ng M, Rudnisky CJ, Dimla B, Tambe E, Nathoo N, et al. Prevalence and severity of diabetic retinopathy in Northwest Cameroon as identified by teleophthalmology. Telemedicine journal and e-health : the official journal of the American Telemedicine Association. 2011;17(4):294-8. |  | √ | -Unclear methodology |
|  | Jolley E, Buttan S, Engels T, Gillani M, Jadoon MZ, Kabona G, et al. Prevalence of Visual Impairment and Coverage of Cataract Surgical Services: Associations with Sex, Disability, and Economic Status in Five Diverse Sites. Ophthalmic epidemiology. 2020;27(6):429-37. |  | √ | -Non-diabetic study participant |
|  | Jonas JB, Sabanayagam C. Epidemiology and risk factors for diabetic retinopathy. Diabetic retinopathy and cardiovascular disease. 27: Karger Publishers; 2019. p. 20-37. |  | √ | -Outcome of interest not reported |
|  | Kaabi YA. Potential roles of anti-inflammatory plant-derived bioactive compounds targeting inflammation in microvascular complications of diabetes. Molecules. 2022;27(21):7352. |  | √ | --Unrelated title |
|  | Kabaso K. Assessment of factors associated with diabetic retinopathy among diabetic patients in Zambia. 2018. |  | √ | -Unrelated title |
|  | Kalaycı M. Causes of Blindness in the Adult Population in Somalia. Turkish journal of ophthalmology. 2020;50(5):288-92. |  | √ | -Unrelated title |
|  | Kaphle D, Gyawali R, Kandel H, Reading A, Msosa JM. Vision Impairment and Ocular Morbidity in a Refugee Population in Malawi. Optometry and vision science : official publication of the American Academy of Optometry. 2016;93(2):188-93. |  | √ | -Non-diabetic study participant |
|  | Katibeh M, Behboudi H, Moradian S, Alizadeh Y, Beiranvand R, Sabbaghi H, et al. Rapid assessment of avoidable blindness and diabetic retinopathy in Gilan Province, Iran. Ophthalmic epidemiology. 2017;24(6):381-7. |  | √ | -Study conducted outside SSA |
|  | Keel S, Lee PY, Foreman J, van Wijngaarden P, Taylor HR, Dirani M. Participant referral rate in the National eye health survey (NEHS). PloS one. 2017;12(4):e0174867. |  | √ | -Unrelated title |
|  | Khajehdehi P, Pakfetrat M, Javidnia K, Azad F, Malekmakan L, Nasab MH, et al. Oral supplementation of turmeric attenuates proteinuria, transforming growth factor-β and interleukin-8 levels in patients with overt type 2 diabetic nephropathy: a randomized, double-blind and placebo-controlled study. Scandinavian journal of urology and nephrology. 2011;45(5):365-70. |  | √ | -Unrelated title |
|  | Khan T, Bertram MY, Jina R, Mash B, Levitt N, Hofman K. Preventing diabetes blindness: cost effectiveness of a screening programme using digital non-mydriatic fundus photography for diabetic retinopathy in a primary health care setting in South Africa. Diabetes research and clinical practice. 2013;101(2):170-6. |  | √ | -Unrelated title |
|  | Killeen AL, Brock KM, Dancho JF, Walters JL. Remote temperature monitoring in patients with visual impairment due to diabetes mellitus: a proposed improvement to current standard of care for prevention of diabetic foot ulcers. Journal of Diabetes Science and Technology. 2020;14(1):37-45. |  | √ | -Unrelated title |
|  | Kisanga SE, Kisanga DH. The role of assistive technology devices in fostering the participation and learning of students with visual impairment in higher education institutions in Tanzania. Disability and rehabilitation Assistive technology. 2022;17(7):791-800. |  | √ | -Unrelated title |
|  | Kizor-Akaraiwe NN, Ezegwui IR, Oguego N, Uche NJ, N Asimadu I, Shiweobi J. Prevalence, awareness and determinants of diabetic retinopathy in a screening centre in Nigeria. Journal of community health. 2016;41:767-71. |  | √ | -Outcome of interest not reported |
|  | Klein R, Klein BE, Kohner EM, Porta M. Saving Sight: A History of Diabetic Eye Disease. Unveiling Diabetes-Historical Milestones in Diabetology. 29: Karger Publishers; 2020. p. 221-41. |  | √ | -Unrelated title |
|  | Köberlein J, Beifus K, Schaffert C, Finger RP. The economic burden of visual impairment and blindness: a systematic review. BMJ open. 2013;3(11):e003471. |  | √ | -Review article |
|  | Kolawole OU, Ashaye AO, Adeoti CO, Mahmoud AO. Survey of blindness and low vision in Egbedore, South-Western Nigeria. West African journal of medicine. 2010;29(5):327-31. |  | √ | -Non-diabetic study participant |
|  | Kolawole OU, Ashaye AO, Mahmoud AO, Adeoti CO. Cataract blindness in Osun state, Nigeria: results of a survey. Middle East African journal of ophthalmology. 2012;19(4):364-71. |  | √ | -Unrelated title |
|  | Korsa AT, Genemo ES, Bayisa HG, Dedefo MG. Diabetes mellitus complications and associated factors among adult diabetic patients in selected hospitals of West Ethiopia. The Open Cardiovascular Medicine Journal. 2019;13(1). |  | √ | -Outcome of interest not reported |
|  | Kropp M, Golubnitschaja O, Mazurakova A, Koklesova L, Sargheini N, Vo T-TKS, et al. Diabetic retinopathy as the leading cause of blindness and early predictor of cascading complications—risks and mitigation. Epma Journal. 2023;14(1):21-42. |  | √ | -Outcome of interest not reported |
|  | Kyari F, Abdull MM, Bastawrous A, Gilbert CE, Faal H. Epidemiology of glaucoma in sub-saharan Africa: prevalence, incidence and risk factors. Middle East African journal of ophthalmology. 2013;20(2):111-25. |  | √ | -Unrelated title |
|  | Kyari F, Abdull MM, Wormald R, Evans JR, Nolan W, Murthy GV, et al. Risk factors for open-angle glaucoma in Nigeria: results from the Nigeria National Blindness and Visual Impairment Survey. BMC ophthalmology. 2016;16:78. |  | √ | -Unrelated title |
|  | Kyari F, Adekoya B, Abdull MM, Mohammed AS, Garba F. The Current Status of Glaucoma and Glaucoma Care in Sub-Saharan Africa. Asia-Pacific journal of ophthalmology (Philadelphia, Pa). 2018;7(6):375-86. |  | √ | -Unrelated title |
|  | Kyari F, Tafida A, Sivasubramaniam S, Murthy GV, Peto T, Gilbert CE. Prevalence and risk factors for diabetes and diabetic retinopathy: results from the Nigeria national blindness and visual impairment survey. BMC public health. 2014;14:1299. |  | √ | -Outcome of interest not reported |
|  | Kyari F, Wormald R, Murthy GV, Evans JR, Gilbert CE. Ethnicity and Deprivation are Associated With Blindness Among Adults With Primary Glaucoma in Nigeria: Results From the Nigeria National Blindness and Visual Impairment Survey. Journal of glaucoma. 2016;25(10):e861-e72. |  | √ | -Unrelated title |
|  | Latorre-Arteaga S, Fernández-Sáez J, Gil-González D. Inequities in visual health and health services use in a rural region in Spain. Gaceta Sanitaria. 2018;32(5):439-46. |  | √ | -Unrelated title |
|  | Lawan A, Mohammed TB. Pattern of diabetic retinopathy in Kano, Nigeria. Annals of African medicine. 2012;11(2):75-9. |  | √ | -Outcome of interest not reported |
|  | Leasher JL, Bourne RR, Flaxman SR, Jonas JB, Keeffe J, Naidoo K, et al. Global estimates on the number of people blind or visually impaired by diabetic retinopathy: a meta-analysis from 1990 to 2010. Diabetes care. 2016;39(9):1643-9. |  | √ | -Review  - |
|  | Lee CS, Zhu S, Wu Q, Hu Y, Chen Y, Chen D, et al. Independent and joint associations of age, pre-pregnancy BMI, and gestational weight gain with adverse pregnancy outcomes in gestational diabetes mellitus. Diabetes Therapy. 2023;14(2):363-75. |  | √ | -Unrelated title |
|  | Lee L, D'Esposito F, Garap J, Wabulembo G, Koim SP, Keys D, et al. Rapid assessment of avoidable blindness in Papua New Guinea: a nationwide survey. The British journal of ophthalmology. 2019;103(3):338-42. |  | √ | -Non-diabetic study participant |
|  | Lee R, Wong TY, Sabanayagam C. Epidemiology of diabetic retinopathy, diabetic macular edema and related vision loss. Eye and vision. 2015;2:1-25. |  | √ | -Outcome of interest not reported |
|  | Lem DW, Gierhart DL, Davey PG. Management of diabetic eye disease using carotenoids and nutrients. Antioxidants—Benefits, Sources, and Mechanisms of Action. 2021. |  | √ | -Unrelated title |
|  | Li AL, Grant D, Gbakie M, Kanneh L, Mustafa I, Bond N, et al. Ophthalmic manifestations and vision impairment in Lassa fever survivors. PloS one. 2020;15(12):e0243766. |  | √ | -Unrelated title |
|  | Li S, Ye E, Huang J, Wang J, Zhao Y, Niu D, et al. Global, regional, and national years lived with disability due to blindness and vision loss from 1990 to 2019: Findings from the Global Burden of Disease Study 2019. Frontiers in public health. 2022;10:1033495. |  | √ | -Unrelated title |
|  | Lin S, Ramulu P, Lamoureux EL, Sabanayagam C. Addressing risk factors, screening, and preventative treatment for diabetic retinopathy in developing countries: a review. Clinical & experimental ophthalmology. 2016;44(4):300-20. |  | √ | - Review article |
|  | Lindfield R, Griffiths U, Bozzani F, Mumba M, Munsanje J. A rapid assessment of avoidable blindness in Southern Zambia. PloS one. 2012;7(6):e38483. |  | √ | -Non-diabetic study participant |
|  | Liu Z, Fu C, Wang W, Xu B. Prevalence of chronic complications of type 2 diabetes mellitus in outpatients-a cross-sectional hospital based survey in urban China. Health and quality of life outcomes. 2010;8:1-9. |  | √ | -Study conducted outside SSA |
|  | Longo-Mbenza B, Muaka MM, Yokobo EC, Phemba IL, Mokondjimobe E, Gombet T, et al. Effects of biomarkers of oxidative stress damage on prevalence and severity of visual disability among black Central Africans. Molecular vision. 2012;18:1619-28. |  | √ | -Unrelated title |
|  | Lott ME, Slocomb JE, Shivkumar V, Smith B, Quillen D, Gabbay RA, et al. Impaired retinal vasodilator responses in prediabetes and type 2 diabetes. Acta ophthalmologica. 2013;91(6):e462-e9. |  | √ | -Unrelated title |
|  | Mabaso R, Oduntan O. Prevalence and causes of visual impairment and blindness among adults with diabetes mellitus aged 40 years and older receiving treatment at government health facilities in the Mopani District, South Africa. African Vision and Eye Health. 2014;73(1):8-15. |  | √ | -Unclear methodology |
|  | Machingura PI, Macheka B, Mukona M, Mateveke K, Okwanga PN, Gomo E. Prevalence and risk factors associated with retinopathy in diabetic patients at Parirenyatwa Hospital outpatients’ clinic in Harare, Zimbabwe. Archives of medical and biomedical research. 2017;3(2):104-11. |  | √ | -Outcome of interest not reported |
|  | Madueña-Angulo SE, Beltran-Ontiveros SA, Leal-Leon E, Contreras-Gutierrez JA, Lizarraga-Verdugo E, Gutierrez-Arzapalo PY, et al. National sex-and age-specific burden of blindness and vision impairment by cause in Mexico in 2019: a secondary analysis of the Global Burden of Disease Study 2019. The Lancet Regional Health–Americas. 2023;24. |  | √ | -Study conducted outside SSA |
|  | Malda MI, Raharjani AS. 4th Global Pediatric Ophthalmology Congress. 2019. |  | √ | -Unrelated title |
|  | Malu KN. Blindness and visual impairment in north central Nigeria: a hospital based study. The Nigerian postgraduate medical journal. 2013;20(2):98-103. |  | √ | -Non-diabetic study participant |
|  | Maluleke KD, Ntimana CB, Mashaba RG, Seakamela KP, Maimela E. Associated factors of diabetic retinopathy in type 1 and 2 diabetes in Limpopo province in South Africa. Frontiers in clinical diabetes and healthcare. 2024;5:1319840. |  | √ |  |
|  | Markos M, Kefyalew B, Tesfaye HB. Pooled prevalence of blindness in Ethiopia: a systematic review and meta-analysis. BMJ open ophthalmology. 2022;7(1). |  | √ | -Outcome of interest not reported |
|  | Martinez-Juarez LA. Assessment of public health services for diabetes mellitus and diabetic retinopathy during the COVID-19 pandemic in the State of Hidalgo, Mexico: London School of Hygiene & Tropical Medicine; 2022. |  | √ | -Unrelated title |
|  | Mashige KP, Ramklass SS. Prevalence and causes of visual impairment among older persons living in low-income old age homes in Durban, South Africa. African journal of primary health care & family medicine. 2020;12(1):e1-e7. |  | √ | -Non-diabetic study participant |
|  | Mathenge W, Bastawrous A, Foster A, Kuper H. The Nakuru posterior segment eye disease study: methods and prevalence of blindness and visual impairment in Nakuru, Kenya. Ophthalmology. 2012;119(10):2033-9. |  | √ | -Unrelated title |
|  | Mathenge W, Bastawrous A, Peto T, Leung I, Yorston D, Foster A, et al. Prevalence and correlates of diabetic retinopathy in a population-based survey of older people in Nakuru, Kenya. Ophthalmic epidemiology. 2014;21(3):169-77. |  | √ | -Outcome of interest not reported |
|  | MBA-Healthcare LGPM. Inside the Eye Disease Just the Facts: A Resource Manual for the Vision Rehabilitation Professionals: Xlibris Corporation; 2020. |  | √ | -Unrelated title |
|  | Mbeboh SN, Christie SA, Carvalho M, Dickson D, Nana T, Embolo F, et al. Prevalence, care-seeking practices and impact of self-reported vision impairment in Southwest Cameroon: a community-based study. BMJ open. 2020;10(11):e041367. |  | √ | -Unrelated title |
|  | McCartney D, Butler JS. Macular pigment and diabetes mellitus. 2020. |  | √ | -Unrelated title |
|  | McInnes A. Diabetes and Mobility Explained: Disease, falls and fractures. |  | √ | -Unrelated title |
|  | Mendonça HR, Carpi-Santos R, da Costa Calaza K, Martinez AMB. Neuroinflammation and oxidative stress act in concert to promote neurodegeneration in the diabetic retina and optic nerve: galectin-3 participation. Neural regeneration research. 2020;15(4):625-35. |  | √ | -Unrelated title |
|  | Mengste YL, Belete GT, Eticha BL, Zeleke TC. Self-Reported Fall-Related Injury and Its Associated Factors among Adults with Visual Impairment Attending St. Paul's Hospital Millennium Medical College, Addis Ababa, Ethiopia. Ethiopian journal of health sciences. 2023;33(2):263-72. |  | √ | -Unrelated title |
|  | Metanmo S, Kuate-Tegueu C, Gbessemehlan A, Dartigues JF, Ntsama MJ, Nguegang Yonta L, et al. Self-reported visual impairment and sarcopenia among older people in Cameroon. Scientific reports. 2022;12(1):17694. |  | √ | -Unrelated title |
|  | Mitchell P, Foran S, Wong T, Chua B, Patel I, Ojaimi E, et al. Guidelines for the management of diabetic retinopathy. National Health and Medical Research Council. 2008. |  | √ | -Unrelated title |
|  | Mohamed SF, Mwangi M, Mutua MK, Kibachio J, Hussein A, Ndegwa Z, et al. Prevalence and factors associated with pre-diabetes and diabetes mellitus in Kenya: results from a national survey. BMC public health. 2018;18:1-11. |  | √ | -Unrelated title |
|  | Momoh ZD, Agweye CT, Oguntolu V, Nkanga D. Diabetic retinopathy screening in Calabar, Nigeria: Factors influencing referrals and uptake of screening service. Nigerian Journal of Ophthalmology. 2017;25(2):118-22. |  | √ | -Unrelated title |
|  | Monaco WA, Crews JE, Nguyen ATH, Arif A. Prevalence of vision loss and associations with age-related eye diseases among nursing home residents aged≥ 65 years. Journal of the American Medical Directors Association. 2021;22(6):1156-61. |  | √ | -Non-diabetic study participant |
|  | Monye HI, Kyari F, Momoh RO. A situational report on low vision services in tertiary hospitals in South-East Nigeria. Nigerian journal of clinical practice. 2020;23(7):919-27. |  | √ | -Unrelated title |
|  | Muaka MM, Longo-Mbenza B. Causes of visual disability among Central Africans with diabetes mellitus. African health sciences. 2012;12(2):193-7. |  | √ | -Unclear methodology |
|  | Muhammad N, Adamu MD, Mpyet C, Bounce C, Maishanu NM, Jabo AM, et al. Impact of a 10-Year Eye Care Program in Sokoto, Nigeria: Changing Pattern of Prevalence and Causes of Blindness and Visual Impairment. Middle East African journal of ophthalmology. 2019;26(2):101-6. |  | √ | -Non-diabetic study participant |
|  | Müller A, Zerom M, Limburg H, Ghebrat Y, Meresie G, Fessahazion K, et al. Results of a rapid assessment of avoidable blindness (RAAB) in Eritrea. Ophthalmic epidemiology. 2011;18(3):103-8. |  | √ | -Non-diabetic study participant |
|  | Muma S, Obonyo S. The prevalence and causes of visual impairment among children in Kenya - the Kenya eye study. BMC ophthalmology. 2020;20(1):399. |  | √ | -Non-diabetic study participant |
|  | Munaw MB, Tegegn MT. Visual impairment and psychological distress among adults attending the University of Gondar tertiary eye care and training center, Northwest Ethiopia: A comparative cross-sectional study. PloS one. 2022;17(2):e0264113. |  | √ | --Non-diabetic study participant |
|  | Mvitu Muaka M, Longo-Mbenza B. Causes of visual disability among Central Africans with diabetes mellitus. African health sciences. 2012;12(2):193-7. |  | √ | -Non-diabetic study participant |
|  | Mwangi N. Diabetic Retinopathy in Kenya: assessment of services and interventions to improve access: London School of Hygiene & Tropical Medicine; 2020. |  | √ | -Unrelated title |
|  | Mwangi N, Bascaran C, Gichuhi S, Kipturgo M, Manyara L, Macleod D, et al. Rationale for integration of services for diabetes mellitus and diabetic retinopathy in Kenya. Eye. 2022;36(Suppl 1):4-11. |  | √ | -Unrelated title |
|  | Mwangi N, Gachago M, Gichangi M, Gichuhi S, Githeko K, Jalango A, et al. Adapting clinical practice guidelines for diabetic retinopathy in Kenya: process and outputs. Implementation science. 2018;13:1-9. |  | √ | -Unrelated title |
|  | Mwangi N, Ng'ang'a M, Gakuo E, Gichuhi S, Macleod D, Moorman C, et al. Effectiveness of peer support to increase uptake of retinal examination for diabetic retinopathy: study protocol for the DURE pragmatic cluster randomized clinical trial in Kirinyaga, Kenya. BMC public health. 2018;18(1):871. |  | √ | -Unrelated title |
|  | Naidoo K, Kempen JH, Gichuhi S, Braithwaite T, Casson RJ, Cicinelli MV, et al. Prevalence and causes of vision loss in sub-Saharan Africa in 2015: magnitude, temporal trends and projections. The British journal of ophthalmology. 2020;104(12):1658-68. |  | √ | -Review |
|  | Nallasamy S, Anninger WV, Quinn GE, Kroener B, Zetola NM, Nkomazana O. Survey of childhood blindness and visual impairment in Botswana. The British journal of ophthalmology. 2011;95(10):1365-70. |  | √ | -Non-diabetic study participant |
|  | Nentwich MM, Ulbig MW. Diabetic retinopathy-ocular complications of diabetes mellitus. World journal of diabetes. 2015;6(3):489. |  | √ | -Unclear methodology |
|  | Zikhali, Thembile, Chester Kalinda, and Zamadonda Nokuthula Xulu-Kasaba. "Screening of diabetic retinopathy using teleophthalmology to complement human resources for eye health: A systematic review and meta-analysis." *Clinics and Practice* 12.4 (2022): 457-467. |  | √ | -Review |
|  | Mathews, Mili Rosline, and S. M. Anzar. "A comprehensive review on automated systems for severity grading of diabetic retinopathy and macular edema." *International Journal of Imaging Systems and Technology* 31.4 (2021): 2093-2122. |  | √ | -Review |
|  | Kibirige, Davis, et al. "Indicators of optimal diabetes care and burden of diabetes complications in Africa: a systematic review and meta-analysis." *BMJ open* 12.11 (2022): e060786. |  | √ | -Review |
|  | Aikaeli, Faith, et al. "Prevalence of microvascular and macrovascular complications of diabetes in newly diagnosed type 2 diabetes in low-and-middle-income countries: A systematic review and meta-analysis." *PLOS global public health* 2.6 (2022): e0000599. |  | √ | -Review |
|  | Curran, Katie, et al. "Inclusion of diabetic retinopathy screening strategies in national-level diabetes care planning in low-and middle-income countries: a scoping review." *Health Research Policy and Systems* 21.1 (2023): 2. |  | √ | -Review |
|  | Maseko, Sharon Nobuntu, Diane van Staden, and Euphemia Mbali Mhlongo. "The rising burden of diabetes-related blindness: A case for integration of primary eye care into primary Health Care in Eswatini." *Healthcare*. Vol. 9. No. 7. MDPI, 2021. |  | √ | -Review |
|  | Njepuome N, Onyebuchi U, Onwusoro M, Igbe M. Visual impairment among public servants in Abuja, Nigeria. The Internet Journal of Ophthalmology and Visual Science. 2012;9(1). |  | √ | -Non-diabetic study participant |
|  | Njikam EJ, Kariuki MM, Kollmann MK, Wilhelm F, Nentwich MM. The magnitude and pattern of diabetic retinopathy in Yaoundé, Cameroon - a cross-sectional hospital-based study. Acta Ophthalmol. 2016;94(2):e156-7. |  | √ | -Outcome of interest not reported |
|  | Nkondi Mbadi AN, Longo-Mbenza B, Mvitu Muaka M, Mbungu FS, Lemogoum D. [Relationship between pulse pressure, visual impairement and severity of diabetic retinopathy in sub-Saharan Africa]. Le Mali medical. 2009;24(3):17-21. |  | √ | -Unclear methodology |
|  | Nkyekyer E. Relationship between concern for vision loss and self-care management in type 1 and type 2 diabetics. 2010. |  | √ | -Unrelated title |
|  | Nnama-Okechukwu CU, Chukwuka PN, Okoye UO. Challenges with Institutional Support Services for Undergraduate Students with Visual Impairment in University of Nigeria Nsukka. Journal of evidence-based social work (2019). 2020;17(6):677-95. |  | √ | -Unrelated title |
|  | Norris KL, Beckles GL, Chou C-F, Zhang X, Saaddine J. Association of socioeconomic status with eye health among women with and without diabetes. Journal of Women's Health. 2016;25(3):321-6. |  | √ | -Unrelated title |
|  | Nwosu SN. Low vision in Nigerians with diabetes mellitus. Documenta ophthalmologica Advances in ophthalmology. 2000;101(1):51-7. |  | √ | -Unclear methodology |
|  | Nyamazana T. The prevalence and management of diabetes mellitus complications at Mankweng Hospital, Limpopo Province 2019. |  | √ | -Outcome of interest not reported |
|  | Nyibong AW. Revalence, Risk Factors and Stage of Diabetic Retinopathy Among Patients Attending the Medical Clinic at Juba Teaching Hospital, South Sudan: University of Nairobi; 2018. |  | √ | -Outcome of interest not reported |
|  | Odugbo OP, Mpyet CD, Chiroma MR, Aboje AO. Cataract blindness, surgical coverage, outcome, and barriers to uptake of cataract services in Plateau State, Nigeria. Middle East African journal of ophthalmology. 2012;19(3):282-8. |  | √ | -Unrelated title |
|  | Oduntan OA, Mashige KP, Kio FE. Colour vision deficiency among students in Lagos State, Nigeria. African health sciences. 2019;19(2):2230-6. |  | √ | -Unrelated title |
|  | Oenga RB. Diabetic Retinopathy; knowledge, attitudes and practice among general practitioners in provincial hospitals in Kenya: University of Nairobi, Kenya; 2012. |  | √ | -Unrelated title |
|  | Ohakosim AN. American Association of Diabetes Educators intervention in managing type 2 diabetes in older home health patients. |  | √ | -Unrelated title |
|  | Olamoyegun M, Ibraheem W, Iwuala S, Audu M, Kolawole B. Burden and pattern of micro vascular complications in type 2 diabetes in a tertiary health institution in Nigeria. African health sciences. 2015;15(4):1136-41. |  | √ | -Unclear methodology |
|  | Olawoye O, Kizor-Akaraiwe N, Pons J, Sarimiye T, Washaya J, Hughes S, et al. Clinical Characteristics and Stage at Presentation of Glaucoma Patients in Sub-Saharan Africa. Journal of glaucoma. 2022;31(9):717-23. |  | √ | -Unrelated title |
|  | Olokoba L, Mahmud O, Adepoju F, Olokoba A. Awareness of diabetic retinopathy among patients with diabetes mellitus in Ilorin, Nigeria. Sudan Journal of Medical Sciences. 2017;12(2):89-100. |  | √ | -Unrelated title |
|  | Olokoba LB. Diabetic retinopathy in Ilorin: a hospital-based study. African journal of medicine and medical sciences. 2019;48(1):63-8. |  | √ | -Outcome of interest not reported |
|  | Omar R, Aziz J. Low vision rehabilitation can improve quality of life. Jurnal Kebajikan Masyarakat. 2010;36:99-110. |  | √ | -Unrelated title |
|  | Teo, Zhen Ling, et al. "Do we have enough ophthalmologists to manage vision-threatening diabetic retinopathy? A global perspective." *Eye* 34.7 (2020): 1255-1261. |  | √ | -Review |
|  | Onakpoya O, Kolawole B, Adeoye A, Okunoye O. Compliance with diabetic retinopathy screening in a Nigerian tertiary hospital. African Journal of Diabetes Medicine. 2015;23(2). |  | √ | -Unrelated title |
|  | Organization WH. Diabetic retinopathy screening: a short guide: increase effectiveness, maximize benefits and minimize harm. 2020. |  | √ | -Unrelated title |
|  | Otoo FS. Nutrients Intake, Antioxidant Micronutrient Status and Vision Disorders among Diabetics Attending Cape Coast Teaching Hospital in the Central Region of Ghana 2017. |  | √ | -Unrelated title |
|  | Rasoulinejad, Seyed Ahmad. "Epidemiological Aspects of Diabetic Retinopathy--A Narrative Review." *Journal of Evolution of Medical and Dental Sciences* 8.43 (2019): 3268-3273. |  | √ | -Review article |
|  | Ovenseri-Ogbomo G, Amiebenomo O, Obahiagbon A. Pattern of referrals by Optometrists in Nigeria in relation to glaucoma and diabetic retinopathy. Journal of Medical and Biomedical Sciences. 2016;5(3):36-44. |  | √ | -Unrelated title |
|  | Ovenseri‑Ogbomo GO. Knowledge of diabetes and its associated ocular manifestations by diabetic patients: A study at Korle‑Bu Teaching Hospital, Ghana. Nigerian Medical Journal. 2013;54(4). |  | √ | -Unrelated title |
|  | Oye J, Mactaggart I, Polack S, Schmidt E, Tamo V, Okwen M, et al. Prevalence and Causes of Visual Impairment in Fundong District, North West Cameroon: Results of a Population-Based Survey. Ophthalmic epidemiology. 2017;24(6):394-400. |  | √ | -Non-diabetic study participant |
|  | Ozawa GY, Bearse Jr MA, Adams AJ. Male–female differences in diabetic retinopathy? Current Eye Research. 2015;40(2):234-46. |  | √ | -Unrelated title |
|  | Pan C-W, Wang S, Qian D-J, Xu C, Song E. Prevalence, awareness, and risk factors of diabetic retinopathy among adults with known type 2 diabetes mellitus in an urban community in China. Ophthalmic epidemiology. 2017;24(3):188-94. |  | √ | -Study conducted outside SSA |
|  | Pan C-W, Wang S, Wang P, Xu C-L, Song E. Diabetic retinopathy and health-related quality of life among Chinese with known type 2 diabetes mellitus. Quality of Life Research. 2018;27:2087-93. |  | √ | -Study conducted outside SSA |
|  | Pan H, Sun J, Luo X, Ai H, Zeng J, Shi R, et al. A risk prediction model for type 2 diabetes mellitus complicated with retinopathy based on machine learning and its application in health management. Frontiers in Medicine. 2023;10:1136653. |  | √ | -Unrelated title |
|  | PAPADAKIS G. Good practices for treating Diabetes Mellitus in a developing country: The University of Athens; 2010. |  | √ | -Unrelated title |
|  | Pappot N, Do NC, Vestgaard M, Ásbjörnsdóttir B, Hajari JN, Lund‐Andersen H, et al. Prevalence and severity of diabetic retinopathy in pregnant women with diabetes—time to individualize photo screening frequency. Diabetic Medicine. 2022;39(7):e14819. |  | √ | -Unrelated title |
|  | Pearce E, Sivaprasad S. A review of advancements and evidence gaps in diabetic retinopathy screening models. Clinical Ophthalmology. 2020:3285-96. |  | √ | -Unrelated title |
|  | Pedersen FN. Diabetic retinopathy as a marker of cognitive dysfunction and depression: a clinical and epidemiological approach. 2023. |  | √ | -Unrelated title |
|  | Peltzer K, Phaswana-Mafuya N. Association between Visual Impairment and Low Vision and Sleep Duration and Quality among Older Adults in South Africa. International journal of environmental research and public health. 2017;14(7). |  | √ | -Unrelated title |
|  | Peng P-H. Assessment the factors associated with the acceptance of retinal screening among patients with diabetes in Taiwan: University of South Carolina; 2010. |  | √ | -Unrelated title |
|  | Pradhan AD, Manson JE, Rifai N, Buring JE, Ridker PM. C-reactive protein, interleukin 6, and risk of developing type 2 diabetes mellitus. jama. 2001;286(3):327-34. |  | √ | -Unrelated title |
|  | Rabiu MM, Taryam MO, Muhammad N, Oladigbolu K, Abdurahman H. Prevalence of diabetes mellitus and diabetic retinopathy in persons 50 years and above in Katsina State Nigeria: a population-based cross-sectional survey. Ophthalmic epidemiology. 2020;27(5):384-9. |  | √ | -Unrelated title |
|  | Ramatea MA, Khanare FP. Improving The well-being of learners with visual impairments in rural Lesotho schools: an asset-based approach. International journal of qualitative studies on health and well-being. 2021;16(1):1890341. |  | √ | -Unrelated title |
|  | Randrianaivo JB, Anholt RM, Tendrisoa DL, Margiano NJ, Courtright P, Lewallen S. Blindness and cataract surgical services in Atsinanana region, Madagascar. Middle East African journal of ophthalmology. 2014;21(2):153-7. |  | √ | -Unrelated title |
|  | Ravi P. Ocular Complications of Uncontrolled Type 2 Diabetes Mellitus and its Management Among Rural Population in and Around Nagamangala Taluk: A Clinical Study: Rajiv Gandhi University of Health Sciences (India); 2018. |  | √ | -Study conducted outside SSA |
|  | Rotimi C, Daniel H, Zhou J, Obisesan A, Chen G, Chen Y, et al. Prevalence and determinants of diabetic retinopathy and cataracts in West African type 2 diabetes patients. Ethnicity & disease. 2003;13(2 Suppl 2):S110-7. |  | √ | -Full text not accessible |
|  | Roy S, Tonkiss J, Roy S. Aging increases retinal vascular lesions characteristic of early diabetic retinopathy. Biogerontology. 2010;11:447-55. |  | √ | -Unrelated title |
|  | Naveen, K. L., Grinton Josvi Veigas, and Ananya Bhattacharjee. "Patient with diabetes mellitus and ocular complications: A brief review." *Asian Journal of Pharmacy and Technology* 11, no. 2 (2021): 141-145. |  | √ | -Review |
|  | Sacks FM, Hermans MP, Fioretto P, Valensi P, Davis T, Horton E, et al. Association between plasma triglycerides and high-density lipoprotein cholesterol and microvascular kidney disease and retinopathy in type 2 diabetes mellitus: a global case–control study in 13 countries. Circulation. 2014;129(9):999-1008. |  | √ | -Unrelated title |
|  | Salami MO, Aribaba OT, Musa KO, Rotimi-Samuel A, Onakoya AO. Relationship between corneal sensitivity and diabetic retinopathy among diabetics attending a Nigerian Teaching Hospital. International ophthalmology. 2020;40:2707-16. |  | √ | -Unrelated title |
|  | Salvetat ML, Pellegrini F, Spadea L, Salati C, Zeppieri M. Non-arteritic anterior ischemic optic neuropathy (NA-AION): a comprehensive overview. Vision. 2023;7(4):72. |  | √ | -Unrelated title |
|  | Samuel SM. Health System Analysis of Diabetes and Diabetic Retinopathy Services in Nigeria–The Case of Akwa Ibom State. 2018. |  | √ | -Unrelated title |
|  | SHofman, K. J., Cook, C., & Levitt, N. (2014). Preventing diabetic blindness: A priority for South Africa. *SAMJ: South African Medical Journal*, *104*(10), 661-662. |  | √ | -Review article |
|  | Senra H, Hernandez-Moreno L, Moreno N, Macedo AF. Anxiety levels moderate the association between visual acuity and health-related quality of life in chronic eye disease patients. Scientific reports. 2022;12(1):2313. |  | √ | -Unrelated title |
|  | Sherwin JC, Dean WH, Metcalfe N. Screening for childhood blindness and visual impairment in a secondary school in rural Malawi. Eye (London, England). 2011;25(2):256-7. |  | √ | -Unrelated title |
|  | Shilpa M. Clinical study of ocular manifestations in patients with type 2 diabetes mellitus: BLDE (Deemed to be University); 2015. |  | √ | -Methodology problem |
|  | Khan, A., et al. "Visual complications in diabetes mellitus: beyond retinopathy." *Diabetic medicine* 34.4 (2017): 478-484. |  | √ | -Review article |
|  | Achigbu, E. O., Agweye, C. T., Achigbu, K. I., & Mbatuegwu, A. I. (2021). Diabetic retinopathy in sub-Saharan Africa: a review of magnitude and risk factors. *Nigerian Journal of Ophthalmology*, *29*(1), 3-12. |  | √ | -Review article |
|  | Khan, A., Petropoulos, I. N., Ponirakis, G., & Malik, R. A. (2017). Visual complications in diabetes mellitus: beyond retinopathy. *Diabetic medicine*, *34*(4), 478-484. |  | √ | -Review article |
|  | Sayin, Nihat, Necip Kara, and Gökhan Pekel. "Ocular complications of diabetes mellitus." *World journal of diabetes* 6, no. 1 (2015): 92. |  | √ | -Review article |
|  | Jingi, A. M., Tankeu, A. T., Ateba, N. A., & Noubiap, J. J. (2017). Mechanism of worsening diabetic retinopathy with rapid lowering of blood glucose: the synergistic hypothesis. *BMC endocrine disorders*, *17*, 1-4. |  | √ | -Review article |
|  | Ruta, L. M., Magliano, D. J., Lemesurier, R., Taylor, H. R., Zimmet, P. Z., & Shaw, J. E. (2013). Prevalence of diabetic retinopathy in Type 2 diabetes in developing and developed countries. *Diabetic medicine*, *30*(4), 387-398. |  | √ | -Review article |
|  | Burgess, P. I., et al. "Epidemiology of diabetic retinopathy and maculopathy in Africa: a systematic review." *Diabetic medicine* 30.4 (2013): 399-412 |  | √ | -Review article |
|  | Ganu, D., N. Fletcher, and N. K. Caleb. "Physical disability and functional impairment resulting from type 2 diabetes in sub-Saharan Africa: a systematic review." *African Journal of Diabetes Medicine* 24.1 (2016). |  | √ | -Review article |
|  | Sileshy B, Hailesilasiie H, Tesfaye Y, Ababu H. Magnitude and associated factors of delirium among patients attending emergency department at Jimma medical center, Jimma, southwest Ethiopia, 2022. BMC psychiatry. 2022;22(1):756. |  | √ | -Unrelated title |
|  | Sivaprasad S, Vasconcelos JC, Prevost AT, Holmes H, Hykin P, George S, et al. Clinical efficacy and safety of a light mask for prevention of dark adaptation in treating and preventing progression of early diabetic macular oedema at 24 months (CLEOPATRA): a multicentre, phase 3, randomised controlled trial. The lancet Diabetes & endocrinology. 2018;6(5):382-91. |  | √ | -Unrelated title |
|  | Sturrock BA, Rees G, Lamoureux EL, Wong TY, Holloway E, Fenwick EK. Individuals' perspectives on coping with vision loss from diabetic retinopathy. Optometry and Vision Science. 2018;95(4):362-72. |  | √ | -Unrelated title |
|  | Sube KL, Lako JD, Mena WG, Seriano OF, Ader AM, Lako RL, et al. Diabetic Retinopathy and the Risk Factors in South Sudan: A Six Months Study. View Article. 2020. |  | √ | -Unclear methodology |
|  | Sundling V. Diabetes eye care in Norwegian optometric practice. 2013. |  | √ | -Unrelated title |
|  | Swami NB, Sanap GS. DIABETIC RETINOPATHY: A SIGHT-THREATENING COMPLICATION OF DIABETES MELLITUS. 2023. |  | √ | -Unclear methodology |
|  | Szymanska M, Mahmood D, Yap TE, Cordeiro MF. Recent advancements in the medical treatment of diabetic retinal disease. International Journal of Molecular Sciences. 2021;22(17):9441. |  | √ | -Unrelated title |
|  | Tabák AG, Herder C, Rathmann W, Brunner EJ, Kivimäki M. Prediabetes: a high-risk state for diabetes development. The Lancet. 2012;379(9833):2279-90. |  | √ | -Unrelated title |
|  | Tafida A, Kyari F, Abdull MM, Sivasubramaniam S, Murthy GV, Kana I, et al. Poverty and Blindness in Nigeria: Results from the National Survey of Blindness and Visual Impairment. Ophthalmic epidemiology. 2015;22(5):333-41. |  | √ | -Unrelated title |
|  | Tang J, Kern TS. Inflammation in diabetic retinopathy. Progress in retinal and eye research. 2011;30(5):343-58. |  | √ | -Unrelated title |
|  | Taryam MO, Rabiu MM, Muhammad N, Oladigbolu K, Abdurrahman H. Prevalence and causes of blindness and visual impairment; and cataract surgical services in Katsina state of Nigeria. The British journal of ophthalmology. 2020;104(6):752-6. |  | √ | -Unrelated title |
|  | Tegegn MT, Assaye AK, Belete GT. Prevalence, causes and associated factors of visual impairment and blindness among older population in outreach site, Northwest Ethiopia. A dual center cross-sectional study. African health sciences. 2023;23(3):683-95. |  | √ | -Non-diabetic study participant |
|  | Thomas BJ, Sanders DS, Oliva MS, Orrs MS, Glick P, Ruit S, et al. Blindness, cataract surgery and mortality in Ethiopia. The British journal of ophthalmology. 2016;100(9):1157-62. |  | √ | -Unrelated title |
|  | Tilahun A, Waqtola C, Tewodros G, Amare D, Yohannis M. Major micro vascular complications and associated risk factors among diabetic outpatients in Southwest Ethiopia. Endocrinol Metab Syndr. 2017;6(4):272. |  | √ | -Outcome of interest not reported |
|  | Tilahun M, Gobena T, Dereje D, Welde M, Yideg G. Prevalence of Diabetic retinopathy and its associated factors among diabetic patients at Debre Markos referral hospital, Northwest Ethiopia, 2019: hospital-based cross-sectional study. Diabetes, Metabolic Syndrome and Obesity. 2020:2179-87. |  | √ | -Outcome of interest not reported |
|  | Tran HM, Mahdi AM, Sivasubramaniam S, Gudlavalleti MV, Gilbert CE, Shah SP, et al. Quality of life and visual function in Nigeria: findings from the National Survey of Blindness and Visual Impairment. The British journal of ophthalmology. 2011;95(12):1646-51. |  | √ | -Unrelated title |
|  | Ugalahi MO, Fasina O, Ogun OA, Ajayi BG. Prevalence of congenital colour vision deficiency among secondary school students in Ibadan, South-West Nigeria. The Nigerian postgraduate medical journal. 2016;23(2):93-6. |  | √ | -Unrelated title |
|  | **Determinants of previous dilated eye examination among type II diabetics in Southwestern Nigeria** |  | √ | -Non-diabetic study participant |
|  | Uzodinma UE, Ede MO, Dike IC, Onah NG, Obiweluozo PE, Onwurah CN, et al. Improving quality of family life among Christian parents of children with low vision in Nsukka catholic diocese using rational emotive family health therapy. Medicine. 2022;101(25):e27340. |  | √ | -Unrelated title |
|  | Valizadeh R, Moosazadeh M, Bahaadini K, Vali L, Lashkari T, Amiresmaili M. Determining the prevalence of retinopathy and its related factors among patients with type 2 diabetes in Kerman, Iran. Osong public health and research perspectives. 2016;7(5):296-300. |  | √ | -Outcome of interest not reported |
|  | Wamucii EG. Self-care knowledge and practices among persons living with Type 2 Diabetes Mellitus attending an outpatient diabetes clinic at Thika level 5 hospital, Kenya: JKUAT-CoANRE; 2023. |  | √ | -Unrelated title |
|  | Wang J, Zhang R-Y, Chen R-P, Sun J, Yang R, Ke X-Y, et al. Prevalence and risk factors for diabetic retinopathy in a high-risk Chinese population. BMC public health. 2013;13:1-7. |  | √ | -Outcome of interest not reported |
|  | Wang Q, Zeng N, Tang H, Yang X, Yao Q, Zhang L, et al. Diabetic retinopathy risk prediction in patients with type 2 diabetes mellitus using a nomogram model. Frontiers in Endocrinology. 2022;13:993423. |  | √ | -Unrelated title |
|  | Kibirige, Davis, et al. "Prevalence and predictors of microvascular and macrovascular diabetes complications in adult Ugandans: a systematic review and meta-analysis." *medRxiv* (2024): 2024-10. |  | √ | -Review |
|  | Cai, K., Liu, Y. P., & Wang, D. (2023). Prevalence of diabetic retinopathy in patients with newly diagnosed type 2 diabetes: A systematic review and meta‐analysis. *Diabetes/metabolism research and reviews*, *39*(1), e3586. |  | √ | -Review |
|  | Azeez, Taoreed Adegoke, et al. "Prevalence and risk factors for diabetic retinopathy in Nigeria: A systematic review and meta-analysis." *The Pan-American Journal of Ophthalmology* 3.1 (2021): 17. |  | √ | -Review |
|  | Lee, Ryan, Tien Y. Wong, and Charumathi Sabanayagam. "Epidemiology of diabetic retinopathy, diabetic macular edema and related vision loss." *Eye and vision* 2 (2015): 1-25. |  | √ | -Review |
|  | Bos, M., & Agyemang, C. (2013). Prevalence and complications of diabetes mellitus in Northern Africa, a systematic review. *BMC public health*, *13*, 1-7. |  | √ | -Review |
|  | Maseko, S. N., van Staden, D., & Mhlongo, E. M. (2021, July). The rising burden of diabetes-related blindness: A case for integration of primary eye care into primary Health Care in Eswatini. In *Healthcare* (Vol. 9, No. 7, p. 835). MDPI. |  | √ | -Review |
|  | Wang Y, Lin Z, Zhai G, Ding XX, Wen L, Li D, et al. Prevalence of and risk factors for diabetic retinopathy and diabetic macular edema in patients with early-and late-onset diabetes mellitus. Ophthalmic research. 2022;65(3):293-9. |  | √ | -Outcome of interest not reported |
|  | Webb EM, Rheeder P, Roux P. Screening in Primary Care for Diabetic Retinopathy, Maculopathy and Visual Loss in South Africa. Ophthalmologica Journal international d'ophtalmologie International journal of ophthalmology Zeitschrift fur Augenheilkunde. 2016;235(3):141-9. |  | √ | -Unrelated title |
|  | Weiserbs KF. The relationship between visual impairment and mortality: The Johns Hopkins University; 1999. |  | √ | -Unrelated title |
|  | Welp A, Woodbury RB, McCoy MA, Teutsch SM, National Academies of Sciences E, Medicine. Understanding the epidemiology of vision loss and impairment in the United States. Making eye health a population health imperative: Vision for tomorrow: National Academies Press (US); 2016. |  | √ | -Study conducted outside SSA |
|  | Wen J, Liu D, Wu Q, Zhao L, Iao WC, Lin H. Retinal image‐based artificial intelligence in detecting and predicting kidney diseases: Current advances and future perspectives. View. 2023;4(3):20220070. |  | √ | -Unrelated title |
|  | Wen L, Wang Y, Lin Z, Wang FH, Ding XX, Li D, et al. The prevalence and causes of visual impairment in type 2 diabetes mellitus in Northeast China. Journal of ophthalmology. 2020;2020(1):5969816. |  | √ | -Study conducted outside SSA |
|  | Williams AS. Visual Impairment in Older Adults. Diabetes Management in Long-Term Settings: A Clinician's Guide to Optimal Care for the Elderly. 2014:195. |  | √ | -Review |
|  | Wong TY, Sabanayagam C. The war on diabetic retinopathy: where are we now? Asia-Pacific Journal of Ophthalmology. 2019;8(6):448-56. |  | √ | -Review |
|  | Yang Z, Tan T-E, Shao Y, Wong TY, Li X. Classification of diabetic retinopathy: Past, present and future. Frontiers in Endocrinology. 2022;13:1079217. |  | √ | -Review |
|  | YINGFENG Z. Epidemiology and Social Determinants of Visual Impairment and Diabetic Retinopathy. 2012. |  | √ | -Study conducted outside SSA |
|  | Yong AC, Ebri AE, O'Connor SE, O'Donovan D, Congdon N, Graham C, et al. Demographic characteristics and ocular needs of children attending child eye clinics in Cross River State, Nigeria: a retrospective analysis of clinical records. BMJ open. 2022;12(5):e060379. |  | √ | -Unrelated title |
|  | Zelalem M, Abebe Y, Adamu Y, Getinet T. Prevalence of visual impairment among school children in three primary schools of Sekela Woreda, Amhara regional state, north-west Ethiopia. SAGE open medicine. 2019;7:2050312119849769. |  | √ | -Non-diabetic study participant |
|  | Zewdu K. Prevalence and associated factors of visual impairment among diabetic patients at Debre Berhan Referral Hospital, North Shoa, Ethiopia, 2017: University of Gondar; 2017. |  | √ | -Non-diabetic study participant |

**List of included studies:**

| **S.N** | **List of studies** | **Remark** | | |
| --- | --- | --- | --- | --- |
|  |  | Included | Excluded | Reason(s) for exclusion |
| 1. | Seid MA, Ambelu A, Diress M, Yeshaw Y, Akalu Y, Dagnew B. Visual impairment and its predictors among people living with type 2 diabetes mellitus at Dessie town hospitals, Northeast Ethiopia: institution-based cross-sectional study. BMC ophthalmology. 2022;22(1):52. | √ |  |  |
| 2. | Asemu MT, Ahunie MA. The impact of diabetes on visual acuity in Ethiopia, 2021. PloS one. 2021;16(8):e0256145. | √ |  |  |
| 3. | Tsegaw A, Alemu S, Dessie A, Patterson CC, Parry EHO, Phillips DIW, et al. Diabetic Retinopathy in Type 2 Diabetes Mellitus Patients Attending the Diabetic Clinic of the University of Gondar Hospital, Northwest Ethiopia. Journal of ophthalmology. 2021;2021:6696548. | √ |  |  |
| 4. | Alemu S, Dessie A, Tsegaw A, Patterson CC, Parry EH, Phillips DI, et al. Retinopathy in type 1 diabetes mellitus: Major differences between rural and urban dwellers in northwest Ethiopia. Diabetes research and clinical practice. 2015;109(1):191-8. | √ |  |  |
| 5. | Demilew KZ, Adimassu NF, Alemu DS. Visual Impairment Among Adult Diabetic Patients and Associated Factors at Debre Brihan Referral Hospital, Central Ethiopia, 2017. Middle East African journal of ophthalmology. 2022;29(1):19-26. | √ |  |  |
| 6. | Alemayehu HB, Tegegn MT, Tilahun MM. Prevalence and associated factors of visual impairment among adult diabetic patients visiting Adare General Hospital, Hawassa, South Ethiopia, 2022. PloS one. 2022;17(10):e0276194. | √ |  |  |
| 7. | Bastola P, Kahsay F, Zewengiel S, Muguleta M. The prevalence of diabetic retinopathy, risk factors, visual impairment and ocular status among patients with diabetes mellitus presenting to Berhan Aini National Referral Hospital, Eritrea: A hospital based study. Journal of Chitwan Medical College. 2016;6(4):37-46. | √ |  |  |
| 8. | Glover SJ, Burgess PI, Cohen DB, Harding SP, Hofland HW, Zijlstra EE, et al. Prevalence of diabetic retinopathy, cataract and visual impairment in patients with diabetes in sub-Saharan Africa. The British journal of ophthalmology. 2012;96(2):156-61. | √ |  |  |
| 9. | Burgess P, Allain T, García‐Fiñana M, Beare N, Msukwa G, Harding S. High prevalence in Malawi of sight‐threatening retinopathy and visual impairment caused by diabetes: identification of population‐specific targets for intervention. Diabetic Medicine. 2014;31(12):1643-50. | √ |  |  |
| 10. | Awadalla H, Noor SK, Elmadhoun WM, Almobarak AO, Elmak NE, Abdelaziz SI, et al. Diabetes complications in Sudanese individuals with type 2 diabetes: Overlooked problems in sub-Saharan Africa? Diabetes & Metabolic Syndrome: Clinical Research & Reviews. 2017;11:S1047-S51. | √ |  |  |
| 11. | Sube KL, Lako JD, Mena WG, Seriano OF, Ader AM, Lako RL, et al. Diabetic Retinopathy and the Risk Factors in South Sudan: A Six Months Study. View Article. 2020. | √ |  |  |
| 12. | Chibuga E. Prevalence, severity, risk indicators and impact of visual impairment among diabetic patients in Mkuranga district, Tanzania: Doctoral dissertation; 2012. | √ |  |  |
| 13. | Seba EG AS, Bwonya BD, Twinamasiko A. Prevalence, risk factors and causes of visual impairment in patients with diabetes at Mbarara Regional Referral Hospital, South Western  Uganda; A hospital based study. 2015 | √ |  |  |
| 14. | Magan T, Pouncey A, Gadhvi K, Katta M, Posner M, Davey C. Prevalence and severity of diabetic retinopathy in patients attending the endocrinology diabetes clinic at Mulago Hospital in Uganda. Diabetes research and clinical practice. 2019;152:65-70. | √ |  |  |
| 15. | Lartey SY, Aikins AK. Visual impairment amongst adult diabetics attending a tertiary outpatient clinic. *Ghana medical journal* 2018;52(2):84-7. | √ |  |  |
| 16. | Lewis AD, Hogg RE, Chandran M, Musonda L, North L, Chakravarthy U, et al. Prevalence of diabetic retinopathy and visual impairment in patients with diabetes mellitus in  Zambia through the implementation of a mobile diabetic retinopathy screening project in the Copperbelt province: a cross-sectional study. *Eye (London, England)* 2018;32(7):1201-8. | √ |  |  |
| 17. | Patel V. Diabetic retinopathy among patients attending university teaching hospitals adult hospital medical clinic in Lusaka: The University of Zambia; 2019. | √ |  |  |
| 18. | Omari S.N. The prevalence and determinants of diabetic retinopathy in Botswana:357  Findings from a screening programme: University of Cape Town; 2017 | √ |  |  |
| 19. | Ayukotang E, Kumah D, Mohammed A. Prevalence of visual impairment among diabetic patients in the Kumba urban area, Cameroon. *International Journal of Innovation and Applied  Studies* 2016;15(4):872 | √ |  |  |
| 20. | Jingi AM, Nansseu JR, Noubiap JJ, Bilong Y, Ellong A, Mvogo CE. Diabetes and visual impairment in sub-Saharan Africa: evidence from Cameroon. *Journal of diabetes and metabolic  disorders* 2015;14:21 | √ |  |  |
| 21. | Onakpoya OH, Adeoye AO, Kolawole BA. Determinants of previous dilated eye examination among type II diabetics in Southwestern Nigeria. *European journal of internal  medicine* 2010;21(3):176-9. | √ |  |  |
| 22. | Onakpoya O, Kolawole B, Adeoye A, Adegbehingbe B, Laoye O. Visual impairment and blindness in type 2 diabetics: Ife-Ijesa diabetic retinopathy study. *International ophthalmology*  2016;36:477-85. | √ |  |  |
| 23. | Sada KB, Sabir AA, Sakajiki AM, Umar MT, Abdullahi U, Sikiru YA. Clinical profile of patients with diabetes mellitus in gusau, Northwestern, Nigeria. *Annals of African medicine*  2021;20(2):78-83 | √ |  |  |
| 24. | Ajayi IA, Raimi TH, Omotoye OJ, Ajite K. Ocular findings in a diabetic retinopathy screening clinic in Southwest Nigeria. *Sky J Med Med Sci* 2016;4:23-7 | √ |  |  |
| 25. | Cleland CR, Burton MJ, Hall C, Hall A, Courtright P, Makupa WU, et al. Diabetic retinopathy in Tanzania: prevalence and risk factors at entry into a regional screening programme. *Tropical Medicine & International Health* 2016;21(3):417-26. | √ |  |  |
| 26. | Mabaso RG, Oduntan OA. Risk factors for visual impairment and blindness amongst black adult diabetics receiving treatment at Government healthcare facilities in Mopani District, Limpopo province, South Africa. *African journal of primary health care & family medicine*  2014;6(1):E1-8. | √ |  |  |
