# Supplementary material for: Prevalence of vision impairment among patients with diabetes mellitus in sub-Saharan Africa: A systematic review and meta-analysis
Source: PLoS One. 2025 Jun 24;20(6):e0326176. doi: 10.1371/journal.pone.0326176 (PMC12186915; doi:10.1371/journal.pone.0326176)
Supplement: Attachment — Submitted filename: Response to Reviewers.docx [file pone.0326176.s010.docx]

**Response to Reviewers**

**Manuscript ID:** PONE-D-24-15939
**Title:** *Prevalence of Vision Impairment among Patients with Diabetes Mellitus in sub-Saharan Africa: a Systematic Review and Meta-Analysis from 2010-2023*.
**Journal:** PLOS ONE

Date; 3-Feb-2025

**Dear Editor and Reviewers,**

Thank you for your support and the opportunity to resubmit our manuscript, "Prevalence of Vision Impairment among Patients with Diabetes Mellitus in sub-Saharan Africa: a Systematic Review and Meta-Analysis from 2010-2023." We sincerely appreciate the detailed comments and helpful suggestions provided. We have worked thoroughly to address all points raised and believe the manuscript has been significantly improved to meet the journal’s standards. We welcome any additional feedback you may have.

We have revised the manuscript accordingly and submitted the required documents, including:

- A **marked-up copy** of the revised manuscript highlighting all changes.
- A **clean version** of the revised manuscript without tracked changes.
- A **point-by-point response** to all comments

Below, you will find our point-by-point responses to the comments. The comments are in black, followed by our responses in blue. All changes made to the original manuscript are highlighted in red within the marked-up copy.

Thank you once again for your guidance and thoughtful consideration.

Kind regards,

Altaseb Beyene Kassaw

On behalf of all the authors.

**Responses to Editor’s Comments**

**1.** Please ensure that your manuscript meets PLOS ONE's style requirements, including those for file naming. The PLOS ONE style templates can be found at…

**Response:** Many thanks for your kind reminder**.** We have carefully reviewed the PLOS ONE style requirements and reformatted our manuscript to ensure full compliance.

**2. [Data Availability Statement]**

**Response**: We apologize for the error in our initial submission. We have now updated the data availability statement to reflect that the data is available. The data can be accessed upon request, and we are prepared to provide it whenever required. Please let us know if any further modifications are needed to meet the journal’s requirements.

**3. -a)** A **numbered table** of all studies identified in the literature search, including excluded studies with reasons for exclusion. A **table of all extracted data** from primary research sources, ensuring that all necessary details are available for replication].

**Response**: We have provided a numbered table of all studies included in the final analysis (Table 1). The PRISMA flow diagram (Figure 1) illustrates the total number of studies identified through the literature search, the study selection process, including the excluded studies and the reasons for their exclusion. However, if this does not fully meet the journal's requirements, we are happy to provide a separate numbered table of all studies identified in the literature search, including those that were excluded from the analysis, along with the specific reasons for their exclusion. Please let us know if you would like us to proceed with this additional table.

b)- **A table of all data extracted from the primary research** sources for the systematic review and/or meta-analysis. The table must include the following information for each study:

Name of data extractors and date of data extraction

Confirmation that the study was eligible to be included in the review.

All data extracted from each study for the reported systematic review and/or meta-analysis that would be needed to replicate your analyses.

**Response**: We have provided a comprehensive table (S6) summarizing all extracted data from the primary research sources in our systematic review and meta-analysis, including the names of data extractors, extraction dates, study eligibility confirmation, and all relevant data required to replicate our analyses.

c) A **table detailing the risk of bias and quality assessments** for each included study.

**Response**: We have included a table that shows the risk of bias and quality assessments for each study included in our analysis (the JBI quality assessment tool /risk of bias assessment tool for prevalence studies (S3)).

**d) An explanation of how missing data were handled.**

**Response**: We appreciate your comment and would like to clarify that as this was a systematic review and meta-analysis, we initially excluded primary studies that did not report the outcome variable, hence there was no missing data that could affect our pooled analysis. However, if we have misunderstood your query, we would be happy to provide further clarification or additional information as required.

**4. [Additional Editor Comments]:**

- Use of English language is poor in certain sections and would require a detailed revision.
- All figures should be attached as separate files in TIF version.
- All supplementary files title/description should be written below reference lists
- All authors’ email except the corresponding author should be removed from the main manuscript.
- The author should follow uniform font size, font type, paragraphing, etc.
- Follow the standard binomial nomenclature, italize journal name and the word ‘et al’

**Response:** we appreciate your kind reminder, too. To address your comments:

1. We have carefully revised the manuscript to improve the English language in the identified sections.
2. All figures have been reattached as separate TIF files as per your request.
3. The titles/descriptions of all supplementary files have been moved to below reference list.
4. We have removed all authors’ emails except for the corresponding author from the main manuscript.
5. We have ensured consistency in font size, font type, and paragraph formatting throughout the manuscript.
6. We have, italicized the word ‘et al.'

**Response to Reviewers’ Comments**

**Reviewer 1**

**[General Assessment]:** The manuscript addresses an important and relevant topic in diabetes management, providing potential contributions to consider optimal control of sugar control to reduce visual impairment as a main complication. However, the significant spelling and grammatical errors throughout the manuscript require attention before the manuscript can be considered for publication

**Response**: Thank you for your thoughtful review of our manuscript. We appreciate your positive feedback on the relevance of the topic and acknowledge your concern about the spelling and grammatical errors throughout the manuscript. We apologize for these issues and have thoroughly revised the manuscript to correct all errors and improve clarity. We believe these revisions have enhanced the quality of the manuscript, and we are grateful for your valuable suggestions.

[**Abstract**]:
Results
The overall pooled prevalence of visual impairment was found to be 29.1% (95% CI: 22%- 36.2%).
The overall prevalence in the forest plot is 29%. Why did you say 29.1% in the abstract?? Please countercheck.
Introduction
Our goal in performing this systematic review and meta-analysis is there for to offer a comprehensive picture of the prevalence of visual impairment and its causes in patients with DM, which will help guide the development of evidence-based management and preventive plans.
Line 88: Please correct the spelling error: “ there for” to therefore.

**Response**: Thank you for your kind reminder. We apologize for the discrepancy between the forest plot and the result in the abstract regarding the pooled prevalence. Upon rechecking, we corrected to 29%.

**Introduction** [Spelling Error]: Line 88: Please correct the spelling error: “ there for” to therefore.

Response: Thank you for pointing out this. We have corrected "there for" to “therefore” in the revised manuscript.

**[Methods]**

Can you clarify, why you only consider studies from 2010-2023?

Eligibility Criteria

Why did you include master's thesis, and dissertations? I think It is less frequently considered in systematic reviews and meta-analyses. Did carefully evaluate their quality and relevance due to potential issues like lack of peer review?

Language: Did you exclude several articles Articles published in other languages?

How did you handle studies reporting visual impairment that was diagnosed concurrently with diabetes? Did you encounter this kind of study during the screening?

**Response**: Thank you for your thoughtful and insightful questions. We appreciate your careful review of our manuscript.

- **Time Frame (2010-2023)**: We limited the inclusion of studies to those published in this year's interval to ensure the relevance and currency of the data, as we aimed to provide an up-to-date overview of the prevalence of visual impairment in DM patients. This time frame was chosen to reflect recent burdens and to ensure the inclusion of studies that are more relevant to current clinical practices and research. Additionally, we observed that there were fewer studies published before 2010, and the majority of relevant research falls within the 2010-2023 period.
- **Eligibility Criteria**
  - We recognize that these sources may not have undergone peer review; however, we carefully assessed their quality and relevance by evaluating the methodology, data quality, and outcomes reported. We included only those that met our eligibility criteria for methodological rigor.
  - Language- Due to resource limitations for translation, we initially applied a filter in each database to include only articles published in English. As a result, articles in other languages were excluded from the review.
  - During the screening process, we did not encounter any studies that clearly reported visual impairment diagnosed concurrently with diabetes. As a result, we did not need to address this scenario in our review.

**[Result]**

Characteristics of the Included Studies

A total of 11240 participants were represented by 26 included studies published from 2010 to 2022. I think 2022 should be 2023. Please counter check

Six studies were conducted in Ethiopia (16, 18-22) and foure 182 in Nigeria(23-26), (Table 1). ‘’Foure’’ should be changed to’’ four’’

Table 1. Summary of 25 studies included in the meta-analysis of the prevalence of Vi among diabetes mellitus patients in Sub-Saharan Countries, 2022.

Vi should be changed to VI. If possible, please kindly avoid the use of abbreviations in Figure and Table titles. Remove 2022 in the title as well.

Aprivations: CS= cross-sectional, T1DM = Type 1 diabetes mellites, T2DM= Type 2 diabetes mellites: Please correct the spelling errors

3.3. Prevalence of Visual Impairment in Diabetes Mellitus

A total of 3,224 visualy impaired diabetic patie were identified from a total of 11, 240 194 participants in 26 included studies The combined meta-analysis of VI in DM patients was found 195 to be 29.1% (95% CI: 22%- 36.2%). Please correct the spelling and punctuation error in this statement.

As a result, the all over prevalence of moderate VI was 15.7% ( 95% CL: 11.7%-20.10%, I 2 199 =98.85%, p-value. Please correct the spelling error. I think the most common terminology is confidence interval, not confidence level

Please have the spacing in the title of the forest plot figure.

Fig.4. Forest plot depicting the allover prevalence of sever visual impairment among diabetes 212 mellitus patients in Sub-Saharan Africa, 2023

Pls check the spelling error of severe and change allover to overall.

When the individual studies were omitted, the pooled estimated prevalence falls within the 95 % CI of the total effect size . Our pooled estimated prevalence of VI varied between 27.29% and 33.5%) after deletion of a single study (Supplementary file 5).

Please correct as follows:

When the individual studies were omitted, the pooled estimated prevalence fell within the 95 % CI of the total effect size. Our pooled estimated prevalence of VI varied between 27.29% and 33.5%) after the deletion of a single study (Supplementary file 5).

It will be good if you manage to anayze the disparities in visual impairment between type I and type 2 Diabetes for studies reporting the prevalence of visual impairment in both types of diabetes

3.4. Causes of Visual Impairment

. Accordingly, the most caus of VI was 245 found to be diabetic retinopathy, representing 35%( 95% CL: 16, 35) followed by cataract 246 34%(95% CL: 21.0, 48.0) and diabetic maculopathy representing 26.0% ( 95% CL: 11.0, 41) (supplementary file 5). Please correct the spelling error ‘Cause ‘.

**Response**: Thank you for your detailed and helpful feedback. We have carefully reviewed your comments and made the necessary corrections, as follows:

1. You are correct that the last study in our review was published in 2023, and we corrected it accordingly.
2. We have corrected the spelling of “foure” to “four”
3. **Table 1 Title**: We have changed “Vi” to visual impairment and removed the abbreviation, as suggested. We also took care to avoid abbreviations in other figure and table titles.
4. We have corrected the spelling of the abbreviations as follows
5. We have corrected the spelling errors in this section and revised the punctuation as follows:
6. We have corrected “confidence level “ to confidence interval ( CI)
7. We have adjusted the spacing in the title of the forest plot figure.
8. We have corrected the spelling of “allover” to “overall” in the figure title.
9. We have revised the sentence “ When the individual studies were omitted, the pooled estimated prevalence……………….”, based the suggestion .
10. We agree that analyzing the disparities in visual impairment between Type 1 and Type 2 Diabetes would provide additional valuable insights. But the data is
11. We have corrected the spelling of “caus” to “cause” in the Causes of Visual Impairment section

**[Discussion]**
Can you look for similar reports in Western countries for comparison?
• What is the plausible explanation for the highest prevalence of visual impairment in Sudan?
References
Some of the reference lists did not have the complete list of information required for the referencing style you used. Please review and revise it accordingly.

**Response**:

- We acknowledge the importance of comparing our findings with similar reports from other regions. However, we encountered challenges in finding directly comparable studies. While we did identify one study, it specifically assessed visual impairment among patients with diabetic retinopathy, which differs from the broader focus of our study.
- We have reviewed and revised the reference list to ensure completeness and adherence to the required reference style.

**Reviewer 2**

**[Major comments]**:

You report that “the majority (of reviewed studies) were followed hospital-based cross-sectional study designs”. This could be a major limitation to the study, considering the difficulties to generalize hospital-based results to population level. This could also explain the particularly high prevalence of visual impairment in the diabetic patient samples.

There is no description of how visual acuity has been measured in each study, whether it was best-corrected visual acuity, and whether there were significant differences in the measurements between studies. This could be a second major limitation to the study.

There are also multiple grammatical errors and typos throughout the text. I highly recommend re-checking the manuscript thoroughly for these and consult an English language expert if needed.

**Response**: Thank you very much for your critical comment. We found your comments and concerns are very insightful and valuable.

- We acknowledge that hospital-based cross-sectional studies may limit the generalizability of our findings to the broader population. To address this concern, we have stated this as a limitation in the discussion section. Additionally, we clarify that almost all the reviewed studies followed an institutional-based cross-sectional design. However, one study did not specify its study design, despite being conducted in an institutional setting.
- Most of the included studies did not specify whether they reported best-corrected visual acuity or uncorrected visual acuity. However, almost all studies used the WHO classification of visual impairment. Since the WHO classification is based on the best-corrected visual acuity in the better-seeing eye, it is reasonable to assume that best-corrected visual acuity was the primary measure in these studies. However, we have now discussed this as a limitation in the Limitations section, acknowledging that differences in visual acuity measurement techniques may have introduced variability in the results.
- We have thoroughly reviewed the manuscript and corrected grammatical errors and typos.

**[Minor comments]:**

Consider removing the WHO classification of visual impairment from the Introduction as it is more properly included in the Materials and Methods.

Uniform the presentation of the data in Table 1 for better clarity.

Recent population-based studies have reported a significant decrease in visual impairment among diabetic patients (see references below). Considering the differing results in the current study, you should add more discussion about this difference.

Laatikainen et al. 2016 Improving visual prognosis of the diabetic patients during the past 30 years based on the data of the Finnish Register of Visual Impairment

Purola et al. 2022 Changes in Visual Impairment due to Diabetic Retinopathy During 1980–2019 Based on Nationwide Register Data

**Response**: Thank you for your helpful suggestions.

- As per your recommendation, we have removed the WHO classification of visual impairment from the Introduction and relocated it to the Materials and Methods section.
- We have revised Table 1 to ensure a more consistent and clear presentation of the data. If there is an additional concern, we are ready to revise a gain.
- We appreciate your insightful comments and the references provided. We have reviewed the findings of the recent population-based studies by *“Laatikainen et al. (2016)”* and “ *Purola et al. (2022)”,* which report a significant decline in visual impairment due to diabetic retinopathy among diabetic patients over the past decades. However, we encountered challenges in directly comparing our results with these studies, as they are primary studies and are based on data from a single cohort in the “Finnish Register of Visual Impairment” from Finland. In contrast, our study is a meta-analysis of data from multiple low-income countries.

But , if the reviewer feels it is appropriate and relevant to our study, we are open to revise the discussion accordingly.
