# Supplementary material for: Prevalence of vision impairment among patients with diabetes mellitus in sub-Saharan Africa: A systematic review and meta-analysis
Source: PLoS One. 2025 Jun 24;20(6):e0326176. doi: 10.1371/journal.pone.0326176 (PMC12186915; doi:10.1371/journal.pone.0326176)
Supplement: Attachment — Submitted filename: Review report.docx [file pone.0326176.s011.docx]

Dear Editor,

Thank you for your invitation to review the manuscript titled “Prevalence of Vision Impairment among Patients with Diabetes Mellitus in Sub-Saharan Africa: A Systematic Review and Meta-Analysis from 2010-2023.” I have provided my comments for each section of the paper.

**Title**: what is the importance of “2010-2023”…………better to mention it in the methodology part

**Abstract: I** found it interesting.

**Introduction**:

Refine the language like: Although several primary studies have revealed that the burden of VI among DM patients is high, their results have revealed substantial variation regarding its prevalence in SSA.. not good sentence,…..and the like.

Moreover, the majority of these research were single-centered, had small sample sizes, and were institution based……………can’t be a reason to carry out your study. See it again.

**Method:**

- I found this section very interesting. The authors clearly apply every step of the PRISMA guideline with in the main document and in the supplementary materials.
- You have used JBI method since your study is a prevalence.

Comments:

- Write the eligibility criteria in paragraph form
- Operationalize some outcome variables

**Results:**

I found it interesting, but refine the language, example

“A significant heterogeneity was seen in the primary studies that were incorporated into this systematic review and meta-analysis. Therefore, to look into the sources of heterogeneity, a sub- group analysis based on the country where the study was conducted was carried out.”……..re-write.

**Discussion part:**

- Generally shallow, fragmented ideas(flowless), lack of art of article writing, not supported by evidences………..make it somewhat deep and keep the flow and support with evidence. This part needs major revision.

**Comments:**

1. Some of the sentences in the first paragraph are not necessary, example The search was limited to articles published between 2010 and 2022, with a total of 26 articles published in the last thirteen years……..already mentioned earlier.
2. I didn’t see the importance of the first paragraph, better to star from the result and support it to others evidence
3. Some conclusions like the following in the discussion part are questionable:

“The reported prevalence of VI in DM patients ranged from 2.7% in Botswana (16) to 78.0% in Ethiopia (15) among the eligible studies, and this meta-analysis indicated that the pooled prevalence of VI in DM patients was 29% (95% CI: 22%- 35%). This suggests that there is a high prevalence of VI in diabetes mellitus patients which indicates poor control of diabetes mellitus-related complications. ……………does it an indication for high prevalence, I don’t think. Instead it indicates for high variability for prevalence and can be used for the justification of conducting this study.

Still, I am not happy with the discussion. The primary outcome (this meta-analysis indicated that the pooled prevalence of VI in DM patients was 29% (95% CI: 22%- 35%). ) must be discussed in detail…………yes the prevalence is high, but to say high and low there must be a comparison. Try to compare it with other studies conducted in northern Africa, Europe………America, Asia………..somewhere else.

“A meta-analysis of all population based studies available worldwide from 1990 to 2010 (42), showed that the prevalence of moderate to severe visual impairment in diabetic patients was 1.9 % globally, 2.8 % in Central Asia, and 2.5% in Central Europe”………discuss it to the main result……...then you can compare and contrast

4. In the sub group analysis, you tried to justify prevalence variability among different countries, and this could be difference in population prevalence of DM in the respective countries , case definition and quality of chronic disease care services, sample size,………and others………no evidences………support with evidences and make them detail. Example you said sample size, but how this sample size varies between the metioned countries and leads this variability in the sub group analysis?
5. Art of article report writing, example, “This study also tried to elucidate the causes of VI in patients with diabetes mellites”……..use other form like in the current study, the common causes of VI were found to be diabetic retinopathy(35%( 95% CI: 16, 35) followed by cataracts 34%(95% CI: 21.0, 48.0), and diabetic maculopathy(26.0% ( 95% CI: 11.0, 41)……….similarly there are many errors of arts of writing like As studies report, Diabetic retinopathy is the most prevalent cause of visual impairment in patients with diabetes, which is supported by our findings, and it is defined by damage to the blood vessels in the retina…………..not a good sentence…………..many ……., and many errors……….the scientific evidences must be addressed in a good way. “The significance of early detection and care of these disorders in preventing vision impairment has been underscored by recent studies (45). Improvements in imaging techniques, such as fundus photography and optical coherence tomography, have made it easier to identify and track these disorders early on, improving patient outcomes (46)”…….seems direct copy

**Final decision: accept with major revision**
