# Supplementary material for: Prevalence of vision impairment among patients with diabetes mellitus in sub-Saharan Africa: A systematic review and meta-analysis
Source: PLoS One. 2025 Jun 24;20(6):e0326176. doi: 10.1371/journal.pone.0326176 (PMC12186915; doi:10.1371/journal.pone.0326176)
Supplement: Attachment — Submitted filename: Response to Reviewer.docx [file pone.0326176.s012.docx]

**Response to Reviewers**

**Dear Editor and Reviewers,**

Thank you again for your continued consideration of our manuscript, *“Prevalence of Vision Impairment among Patients with Diabetes Mellitus in sub-Saharan Africa: a Systematic Review and Meta-Analysis from 2010-2023” (PONE-D-24-15939R1).* We appreciate the opportunity to revise and resubmit it for further evaluation.

We have carefully reviewed the additional comments and have made the necessary revisions to address the remaining points. We believe that these changes have further strengthened the manuscript, and we believe that it now fully meets PLOS ONE's publication criteria.

In this resubmission, you will find the following:

- A detailed rebuttal letter **(Response to reviewers)**. We outline the actions taken to incorporate these suggestions and offer clear explanations.
- A marked-up copy of the manuscript **(Revised manuscript with track changes):** This version highlights all the changes made to the manuscript.
- An unmarked version of the revised paper **(Manuscript):** This is a clean version of the manuscript reflecting all the revisions.

We believe the current version of the manuscript reflects improvements and addresses the outstanding issues. We remain fully committed to responding to any additional feedback, should further clarification or revisions be required.

Thank you again for your time and guidance in the review process.

Sincerely,

Altaseb Beyene Kassaw

On behalf of all co-authors

E-mail: altasebbeyene7@gmail.com

**Response to Reviewers’ Comments**

**Reviewer 2**

**[General comment]**

“Thank you for addressing the concerns raised in the last review. Unfortunately, I do not feel that the responses regarding the lack of description of visual acuity measurements and the difficulties to generalize the data to population level are satisfactory, which would be critical considering that both limitations can lead to unacceptable bias for the analyses and conclusions of the study”.

**Response**: We sincerely thank the reviewer’s continued feedback and for highlighting these important concerns. We understand and acknowledge the critical importance of clearly describing visual acuity measurements and addressing generalizability to minimize potential biases.

We have revised the manuscript to provide a more detailed explanation of how visual acuity was measured in our study. These details have been added as supplementary material, **S4**. Specifically, we have now included:

- The method and chart used for measuring visual acuity (e.g., Snellen, ETDRS, or other).
- Whether measurements were taken with best-corrected or presenting visual acuity.

Regarding generalizability, we have added a discussion of these limitations in the revised manuscript.

[**Title**]:

What is the importance of “2010–2023”? … Better to mention it in the methodology part.

**Response: We** thank the reviewer for this helpful observation, and we have revised accordingly**.**

**[Introduction]**:

“Refine the language like: Although several primary studies have revealed that the burden of VI among DM patients is high, their results have revealed substantial variation regarding its prevalence in SSA.. not good sentence,…..and the like.

Moreover, the majority of these research were single-centered, had small sample sizes, and were institution based……………can’t be a reason to carry out your study. See it again”.

**Response:** We thank the reviewer for this valuable feedback. We agree the original introduction needed clearer language. We have revised the paragraph to improve structure and flow. We also strengthened the rationale for this review by highlighting the need for comprehensive synthesis due to methodological heterogeneity and the lack of pooled regional estimates, beyond just study design limitations.

**[Method]:**

- “I found this section very interesting. The authors clearly apply every step of the PRISMA guideline with in the main document and in the supplementary materials.
- You have used JBI method since your study is a prevalence.

Comments:

- Write the eligibility criteria in paragraph form
- Operationalize some outcome variables”

**Response**: We appreciate the reviewer’s positive feedback on the Methods section and are grateful for his/her constructive suggestions.

We have revised the ***eligibility criteria*** subsection to present the inclusion and exclusion criteria in paragraph format, as suggested by the reviewer.

In the revised manuscript, we have added ***operational definitions*** for key outcome variables, such as "vision impairment" and its different levels (e.g., mild, moderate, severe, blindness), based on the definitions used in the included studies. We have specified how these variables were measured and categorized within the context of our review.

**[Result]:**

**Comments**: I found it interesting, but refine the language, example

“A significant heterogeneity was seen in the primary studies that were incorporated into this systematic review and meta-analysis. Therefore, to look into the sources of heterogeneity, a sub- group analysis based on the country where the study was conducted was carried out.”……..re-write.

**[Discussion]**

**Comments:** • Generally shallow, fragmented ideas (flowless), lack of art of article writing, not supported by evidences………..make it somewhat deep and keep the flow and support with evidence. This part needs major revision.

**Response**: We sincerely appreciate the reviewer’s thoughtful and detailed critique of the Discussion section. We acknowledge that this section requires substantial revision to improve its depth, flow, and support with evidence. In response, we have substantially revised the entire Discussion section. We have taken the reviewer's comments seriously and have made significant changes to address the concerns.

**Specific Comments and Responses:**

**Comments: “Some of the sentences in the first paragraph are not necessary, for example, 'The search was limited to articles published between 2010 and 2022, with a total of 26 articles published in the last thirteen years...already mentioned earlier”**

**Response**: We agree that this sentence is redundant. We have removed it from the revised Discussion section.
