# Supplementary material for: Prevalence of vision impairment among patients with diabetes mellitus in sub-Saharan Africa: A systematic review and meta-analysis
Source: PLoS One. 2025 Jun 24;20(6):e0326176. doi: 10.1371/journal.pone.0326176 (PMC12186915; doi:10.1371/journal.pone.0326176)
Supplement: Attachment — Submitted filename: Response to Reviewers,.docx [file pone.0326176.s013.docx]

**Dear Academic Editor and Reviewers**,

Thank you once again for your continued consideration of our manuscript, *“Prevalence of Vision Impairment among Patients with Diabetes Mellitus in sub-Saharan Africa: A Systematic Review and Meta-Analysis from 2010–2023”* (PONE-D-24-15939R1). We sincerely appreciate the opportunity to revise and resubmit our work for further evaluation.

We have carefully reviewed the additional comments and have made the necessary revisions to address all remaining concerns. We believe these changes have significantly strengthened the manuscript and that it now fully meets PLOS ONE’s publication criteria.

General Editorial Comments

1***.*** Intensive proofreading needed (grammar, punctuation, typographical errors).

**Response**: We have carefully revised the entire manuscript for grammar, punctuation, and clarity.

2. Avoid the year interval from the title

**Response**: The title has been revised to remove the year interval. We now indicate the study period (2010–2023) under the Methods section (inclusion/exclusion criteria).

3. Abstract background not explanatory.

**Response**: The background section of the abstract has been revised to provide clearer context regarding the importance of the study and its public health implications.

4. Discussion lacks explanations for variations/similarities and overuses non-SRMA references.

**Response**: Thank you for your insightful comment. Following your suggestion, we have revised the discussion section to include explanatory reasoning for the regional variations and similarities in the prevalence of vision impairment among diabetic patients. While we encountered difficulty getting a sufficient number of regionally comparable similar SRMAs, we have incorporated large-scale nationwide studies to support our comparisons.

5. Avoid asterisks in references.

Response**:** All asterisks have been removed from the reference list.

6. Follow standard binomial nomenclature, italicize journal names and ‘et al.’:

**Response**: We have revised the references to follow standard binomial nomenclature and formatting, including italicizing journal names and ‘et al.’ as required.

We hope the revisions address your concerns and improve the manuscript's clarity and rigor. We appreciate the opportunity to revise and resubmit our work.

Kind regards,

Altaseb Beyene Kassaw

On behalf of all co-authors
